# Individual Variation in Lipidomic Profiles of Healthy Subjects in Response to Omega-3 Fatty Acids 

**DOI:** 10.1371/journal.pone.0076575

**Published:** 2013-10-24

**Authors:** Malin L. Nording, Jun Yang, Katrin Georgi, Christine Hegedus Karbowski, J. Bruce German, Robert H. Weiss, Ronald J. Hogg, Johan Trygg, Bruce D. Hammock, Angela M. Zivkovic

**Affiliations:** 1 Computational Life Science Cluster (CLiC), Department of Chemistry, Umeå University, Umeå, Sweden; 2 Department of Entomology, University of California Davis, Davis, California, United States of America; 3 Comprehensive Cancer Center, University of California Davis, Davis, California, United States of America; 4 Department of Food Science & Technology, University of California Davis, Davis, California, United States of America; 5 Foods for Health Institute, University of California Davis, Davis, California, United States of America; 6 Nephrology Division, Department of Medicine, University of California Davis, Davis, California, United States of America; 7 Medical Service, Sacramento VA Medical Center, Sacramento, California, United States of America; 8 Scott & White Clinic, Temple, Texas, United States of America; 9 Department of Nutrition, University of California Davis, Davis, California, United States of America; Southern Illinois University School of Medicine, United States of America

## Abstract

**Introduction:**

Conflicting findings in both interventional and observational studies have resulted in a lack of consensus on the benefits of ω3 fatty acids in reducing disease risk. This may be due to individual variability in response. We used a multi-platform lipidomic approach to investigate both the consistent and inconsistent responses of individuals comprehensively to a defined ω3 intervention.

**Methods:**

The lipidomic profile including fatty acids, lipid classes, lipoprotein distribution, and oxylipins was examined multi- and uni-variately in 12 healthy subjects pre vs. post six weeks of ω3 fatty acids (1.9 g/d eicosapentaenoic acid [EPA] and 1.5 g/d docosahexaenoic acid [DHA]).

**Results:**

Total lipidomic and oxylipin profiles were significantly different pre vs. post treatment across all subjects (*p*=0.00007 and *p*=0.00002 respectively). There was a strong correlation between oxylipin profiles and EPA and DHA incorporated into different lipid classes (r^2^=0.93). However, strikingly divergent responses among individuals were also observed. Both ω3 and ω6 fatty acid metabolites displayed a large degree of variation among the subjects. For example, in half of the subjects, two arachidonic acid cyclooxygenase products, prostaglandin E_2_ (PGE_2_) and thromboxane B2 (TXB_2_), and a lipoxygenase product, 12-hydroxyeicosatetraenoic acid (12-HETE) significantly decreased post intervention, whereas in the other half they either did not change or increased. The EPA lipoxygenase metabolite 12-hydroxyeicosapentaenoic acid (12-HEPE) varied among subjects from an 82% decrease to a 5,000% increase.

**Conclusions:**

Our results show that certain defined responses to ω3 fatty acid intervention were consistent across all subjects. However, there was also a high degree of inter-individual variability in certain aspects of lipid metabolism. This lipidomic based phenotyping approach demonstrated that individual responsiveness to ω3 fatty acids is highly variable and measurable, and could be used as a means to assess the effectiveness of ω3 interventions in modifying disease risk and determining metabolic phenotype.

## Introduction

The widely reported beneficial effects of ω3 fatty acids (FA) in modifying risk for chronic diseases, particularly heart disease, have recently been called into question. A recent meta-analysis of 20 randomized clinical trials including over 68,000 patients reported no statistically significant effect of ω3 fatty acids either taken as supplements or through diet (largely as fatty fish) on all-cause mortality, cardiac death, sudden death, myocardial infarction or stroke [1]. This result has come as a surprise after several decades of reports of beneficial effects, starting with the early studies in the 1970s that found low rates of heart disease in Inuit populations with high ω3 diets [2]. Conflicting reports arose as early as 9 years ago, when the DART-2 trial, discovered an apparent harmful effect of ω3 taken as fish oil supplements [3]. Subsequently, several insightful reviews and commentaries have highlighted the need to personalize dietary interventions such as ω3 fatty acid intake to individual needs and to consider the broader context of the overall diet and environment [4,5]. The question remains, if it is indeed true that different groups of individuals respond differently to ω3 FA, can these differences in response be measured and quantified in order to enable a more personalized approach?

 There is evidence that combined intakes of 250-500 mg/d of eicosapentaenoic acid (EPA; 20:5n3) and docosahexaenoic acid (DHA; 22:6n3), the two main long-chain ω3 FA that are associated with most of the bioactivity of ω3s, significantly reduce the risk of fatal coronary heart disease, primarily through the prevention of cardiac arrhythmias [6]. Other potential mechanisms by which ω3 FA may be reducing heart disease risk include lowering plasma triglycerides [7], modifying lipoprotein particle size, decreasing blood pressure, as well as inhibiting platelet aggregation [8]. It is universally agreed that ω3 FA decrease the production of VLDL, thereby reducing plasma triglycerides by as much as 30-50% [9]. The effects on lipoprotein particle distribution, including a decrease in VLDL size, increases in LDL and HDL size, and increases or decreases in LDL and HDL cholesterol are inconsistent and may be due to differences in the specific effects of the individual long chain ω3 EPA and DHA [10-12] as well as genetic factors [13], however, the variability in these responses has not been well characterized to date. Additionally, ω3 FA have been implicated in decreasing inflammation via direct effects on lipid signaling mediators, or oxylipins. ω3 FA decrease production of several pro-inflammatory ω6 arachidonic acid (ARA, 20:4n6) derived eicosanoids including the cyclooxygenase (COX) product prostaglandin E2 (PGE_2_) [14] and the lipoxygenase (LOX) product leukotriene B4 (LTB_4_) both in vivo and in vitro [14-18]. The doses of EPA and DHA that are associated with all of the above effects (decreasing triglycerides, modifying lipoprotein particle distribution, and anti-inflammatory effects) are typically much higher than the 250-500 mg/d dose needed for anti-arrhythmia effects and are in the range of 1-5 g/d EPA+DHA.

 We hypothesized that a comprehensive lipidomic approach is necessary to determine the overall effects of ω3 fatty acids in individuals. We further hypothesized that a comprehensive lipidomic approach for measuring response to ω3 fatty acids would reveal both those aspects of response that are consistent and those that vary among individuals. Recently, the effects of ω3 FA on partial plasma lipidomes have been published, including a report on fatty acids and lipid classes [19], studies on oxylipins in healthy subjects [20], in patients with IgA nephropathy [21], and in patients with asthma [22], and a number of publications on the effects on lipoproteins (reviewed in 11,23-26). However, the effects on the comprehensive lipidomic profile including fatty acids, lipid classes, lipoprotein distribution, and oxylipins have not been examined simultaneously to date. In this study, we examined the comprehensive lipidomic profiles in 12 healthy subjects pre vs. post 6 weeks of administration of ω3 FA taken as fish oil delivering 1.9 g/d EPA + 1.5 g/d DHA. 

## Methods

### Subjects

This was an open label, single arm intervention pilot study completed during the summer of 2008 to investigate the ability of comprehensive lipid profiling to discern both the variable and consistent aspects of lipid metabolic responsiveness to ω3 FA in healthy subjects. Healthy subjects were recruited on the University of California Davis campus by flyering and announcements at seminars. They were healthy adults with the following mean (± SD) characteristics: age, 32 ± 8 y; weight, 67 ± 17 kg; body mass index (BMI), 23 ± 3 kg/m^2^. Exclusion criteria were age < 18 or > 65, pregnancy or nursing, any existing medical condition/disease diagnosis, BMI < 18 or > 30, anemia and/or conditions that would influence ability to donate blood safely, and recently recovering from an illness, injury, or infection. The subjects ate their regular diets throughout the study except they abstained from eating seafood or supplements containing ω3 FA. The subjects were administered 6 g/d fish oil (Ocean Nutrition, lot 18394) containing ω3 FA (1.9 g/d EPA + 1.5 g/d DHA) in capsule form, representing 32% and 25% of FA, as provided in the certificate of analysis. The FA composition of the fish oil capsules (data provided by manufacturer) was as follows: 35% 20:5n3, 26% 22:6n3, 2% 18:1n9, 11% 20:1n9, 2% 20:4n6, 2% 20:4n3, 6% 22:1n11, 1% 22:1n9, 1% 21:5n3, 6% 22:5n3, 1% 24:1n9, and about 6% of other minor FA each at less than 1% concentration.

The subjects came to the Ragle Center on the University of California campus on three occasions. On the first visit, study personnel screened and consented the subjects. Subjects filled out a questionnaire about their health and current medication use to determine whether they met inclusion criteria (weight at least 49 kg, adult (aged 18-65 years old), disclose which medications currently taking, able to come to the Ragle Center at the designated time, able to give blood, able to take 6 g fish oil per day for 6 weeks, able to carry out first morning urine collection, able to stop or avoid taking NSAIDs and allergy medications for 6 weeks, able to stop or avoid eating seafood and seaweed for 6 weeks). At the baseline and post-intervention visits fasting blood samples were collected by venipuncture after a 12-hour overnight fast and height and weight were recorded. Blood was collected using BD Vacutainer lavender-top EDTA tubes. Whole blood was centrifuged in a tabletop ultracentrifuge for 10 min at 4°C at 13,000 rpm within 15 min of collection. The plasma was separated into 1.5-mL aliquots and immediately frozen at -70°C until analysis. Subjects also collected first morning urine samples however urine analysis results are not reported here.

Study personnel administered 6 weeks of fish oil capsules (each week’s supply of capsules in an individual bottle) at the baseline visit. Subjects were instructed to take 6 capsules each day at their convenience at home/work, with a suggestion to take the capsules at the same time each day, preferably in the evening after the last meal of the day. After 6 weeks the subjects returned for the post intervention visit at which time another fasting blood draw was collected and subjects’ weight and height was measured. Subjects kept 3-day diet records at the beginning and end of the study. Subjects were compensated for their time with gift cards and were also given their baseline blood lipid results. Study personnel contacted subjects by e-mail and/or phone throughout the 6 weeks of the study to ensure compliance and to remind subjects about their post-intervention visit. Twenty-three subjects were screened for eligibility, 6 of whom were taking medications and/or were not willing to abstain from taking medications, and 4 of whom declined to be enrolled. Thirteen subjects were enrolled with one subject dropping out due to gastrointestinal discomfort (diarrhea) after the first day of the study. Twelve subjects (8 female, 4 male) completed the study and were included in the analysis. Two of the female subjects reported that their menstrual cycles were significantly longer than usual during the course of the intervention but there were no reports of adverse events and the fish oil was well tolerated. The subject characteristics are summarized in Table **1**. Sample size calculations were not possible given the exploratory nature of the study. 

**Table 1:** Subject characteristics^1^.

**Subject ID**	**202**	**203**	**206**	**207**	**212**	**214**	**218**	**220**	**222**	**225**	**227**	**231**	**Mean (SD)**
Age	26	31	33	32	26	21	35	55	28	34	32	28	31 (8.)
Gender	F	F	F	F	M	F	M	M	F	M	F	F	–
Height (m)	1.75	1.57	1.63	1.73	1.75	1.63	1.83	1.70	1.60	1.91	1.55	1.63	1.7 (0.1)
Weight (kg)	66	49	69	64	80	55	94	65	53	97	53	50	66 (17)
BMI (kg/m^2^)	22	20	26	21	26	21	28	22	21	27	22	19	23 (3)
Baseline PC EPA%^2^	0.8	0.4	1.0	1.6	1.1	0.2	1.7	0.5	0.6	0.6	0.7	1.0	0.9 (0.5)
Baseline PC DHA%^2^	2.4	3.0	4.0	4.0	4.1	2.1	4.5	1.9	4.4	2.8	4.7	3.1	3.4 (1)
Baseline PC ARA%^2^	6.9	7.5	14.2	10.5	12.1	8.0	11.2	11.3	9.3	9.8	9.2	8.1	9.8 (2.1)
Baseline PC EPA/ARA	0.1	0.2	0.2	0.2	0.2	0.1	0.2	0.1	0.2	0.1	0.2	0.2	0.2 (0.04)
Baseline PE EPA%^2^	0.7	0.5	1.6	0.7	1.7	0.5	2.0	0.4	0.7	1.1	0.8	1.4	1.0 (0.6)
Baseline PE DHA%^2^	5.2	5.4	8.4	6.7	7.7	6.0	7.2	3.3	8.3	7.5	6.5	5.8	6.5 (1.5)
Baseline PE ARA%^2^	23	26	30	26	27	25	28	27	27	27	26	26.9	27 (1.7)
Baseline PE EPA/ARA	0.03	0.02	0.06	0.03	0.06	0.02	0.07	0.01	0.03	0.04	0.03	0.05	0.04 (0.02)

^1^ PC, phosphatidylcholine; EPA, eicosapentaenoic acid; DHA, docosahexaenoic acid; ARA, arachidonic acid; phosphatidylethanolamine.

^2^ Fatty acids are presented as mol % of total fatty acids within each lipid class

#### Ethics Statement

The Institutional Review Board of the University of California Davis approved the study protocol, and written informed consent was obtained from all subjects. The study was conducted according to the principles expressed in the Declaration of Helsinki. This trial was registered at clinicaltrials.gov as NCT01838239.

### Lipid Profiling

Compositional lipid analysis was performed by Lipomics Technologies, Inc. (West Sacramento, CA) according to the method described by Watkins et al [27]. Coefficients of variation for this analysis were reported previously [28]. Briefly, the lipids from plasma (200 µL) were extracted using a modified Folch extraction in chloroform:methanol (2:1 v/v) [29]. Extracted lipids were separated by preparative high-pressure liquid chromatography into 7 lipid classes— free fatty acids (FFA), diacylglycerol (DG), triacylglycerol (TG), cholesterol ester (CE), lysophosphatidylcholine (LY), phosphatidylcholine (PC), and phosphatidylethanolamine (PE). The FA from all lipid classes were trans-esterified in 3 N methanolic HCl. The resulting FA methyl esters were extracted and analyzed by gas chromatography using an Agilent 6890 gas chromatograph (Palo Alto, CA) equipped with a 30-m HP-88 capillary column and a flame-ionization detector. The following FA were quantified in each lipid class and summed in order to obtain total lipid class quantities: 14:0, 15:0, 16:0, 18:0, 20:0, 22:0, 24:0, 14:1n5, 16:1n7, t16:1n7, 18:1n9, t18:1n9, 18:1n7, 18:2n6, t18:2n6, 18:3n6, 18:3n3, 18:4n3, 20:1n9, 20:2n6, 20:3n9, 20:3n6, 20:4n6, 20:3n3, 20:4n3, 20:5n3, 22:1n9, 22:2n6, 22:4n6, 22:5n3, 22:6n3, 24:1n9, and 24:6n3, and the plasmalogen derivatives of 16:0, 18:0, 18:1n9, and 18:1n7 expressed as dm16:0, dm18:0, dm18:1n9, and dm18:1n7 respectively. Each individual FA was quantified as nmol FA/g plasma. Results are expressed as mean ± SD. All univariate and multivariate statistical analyses were performed using the quantitative data. Mol % baseline data are presented in Table 1 and changes in mol % composition of key fatty acids are presented in the results in order to be able to relate these results to published literature, which for the most part reports mol % data.

### Oxylipin Profiling

Oxylipins were analyzed as described previously [30]. Briefly, the plasma samples were subjected to solid phase extraction (SPE) on 60 mg Waters Oasis-HLB cartridges (Milford, MA). The elutions from the SPE cartridges were evaporated using a Speedvac (Jouan, St-Herblain, France) and reconstituted in a 200 nM 1-cyclohexyl ureido, 3-dodecanoic acid (CUDA) in a methanol solution. An Agilent 1200 SL (Agilent Corporation, Palo Alto, CA) equipped with a 2.1 × 150 mm Eclipse Plus C18 column with a 1.8 μm particle size (Agilent Corporation, Palo Alto, CA) was used for liquid chromatography with the following conditions: the autosampler was kept at 4 °C, mobile phase A was water with 0.1% glacial acetic acid, mobile phase B was acetonitrile/methanol (84:16) with 0.1% glacial acetic acid, gradient elution was performed at a flow rate of 250 μL/min. Chromatography was optimized to separate all analytes in 21.5 min according to their polarity with the most polar analytes, prostaglandins and leukotrienes eluting first, followed by the hydroxy and epoxy fatty acids. The column was connected to a 4000 QTrap tandem mass spectrometer (Applied Biosystems Instrument Corporation, Foster City, CA) equipped with an electrospray source (Turbo V). The instrument was operated in negative multiple reaction monitor (MRM) mode. The optimized chromatographic conditions and the MRM transitions, as well as extraction efficiencies and validation (including coefficients of variation) were reported previously [30]. Quality control samples were analyzed at a minimum frequency of 10 hours to ensure stability of the analytical calibration throughout the analysis. Analyst software 1.4.2 was used to quantify the peaks according to the standard curves. A total of 87 oxylipins were measured by this method and are shown in a pathway map (Figure **S1**). 

### Lipoprotein Profiling

Lipoprotein profiling was performed by LipoScience Inc. (Raleigh, NC) using NMR spectroscopy and the LipoProfile-3 algorithm, which included measurement of the following parameters: VLDL & Chylomicron Particles (total), Large VLDL & Chylomicrons Particles, Medium VLDL Particles, Small VLDL Particles, LDL Particles (total), IDL Particles, Large LDL Particles, Small LDL Particles (total), Medium Small LDL Particles, Very Small LDL Particles, HDL Particles (total), Large HDL Particles, Medium HDL Particles, and Small HDL Particles reported in nmol/L; VLDL Size, LDL Size, and HDL Size reported in nm; and Triglyceride (total), VLDL & Chylomicron Triglyceride (total), and HDL Cholesterol (total) reported in mg/dL. The lipoprotein particle concentrations were quantified from the amplitudes of the lipid methyl group NMR signals according to previously published and validated methods [31]. 

### Univariate Statistical Analysis

Data were tested for normality and pre-processed to account for the high data density of the metabolomic approach used in this study as described by Zivkovic et al. [28]. Metabolites with >33% missing observations or that were not detected (i.e. were below level of quantitation) were excluded from analysis. Change-detection plots were used to determine whether the observed signal was greater than that which could be expected by chance (noise). All metabolite data were assessed for normality with histograms. When non-normality was detected, values were log_2_ transformed prior to analysis. The Bonferroni correction was used to correct for multiple testing in this lipidomic data set containing a total of 261 variables. Differences were considered significant at the adjusted level of α ≤ 0.0002. Paired t-tests were conducted to examine the changes in individual metabolites from pre to post intervention. Weight-adjusted EPA and DHA dose were calculated as the total amount of EPA and DHA in the daily supplement respectively, divided by each subject’s weight in kg. Percentage change (% change) was calculated as (Post-Pre)/Pre * 100. Standard least squares linear regressions were run for each metabolite adjusting for age, gender, body weight, and BMI. Pearson’s correlations were performed to assess correlations between 1) baseline values and % change in all of the metabolites, 2) % change in the supplemented FA (EPA and DHA) and age, body weight, BMI, and 3) % change in oxylipin precursors (ARA, EPA, and DHA) and % change in oxylipin products and lipoprotein particle numbers and sizes. T-tests were performed to assess differences in % change in metabolites between male subjects and female subjects. Diet records were analyzed by Nutrihand software (Nutrihand Inc., Soraya,CA). Diet records were imported into the software and average values for macronutrients were calculated for all subjects with available records. Values for the week prior to beginning the intervention and at the end of the intervention were compared by paired t-test. All statistics were performed using Microsoft Excel and JMP statistical software (SAS Institute Inc., Cary, NC).

### Multivariate Statistical Analysis

Multivariate statistical analysis was performed using several different methods, all available in the software SIMCA 13 (Umetrics, Umeå, Sweden). Principal component analysis (PCA) was used to identify patterns in the data [32]. Regression analysis in terms of orthogonal projections to latent structures discriminant analysis (OPLS-DA) was used to investigate relationships in the data [33]. Orthogonal 2 projections to latent structures (O2PLS) was used to investigate co-dependencies in the data [34]. In applying these methods, the complexity of the data set was reduced, thereby facilitating interpretation of the variability in the lipidomic profile. This was done by extracting systematic variability into model components, plotted against each other. The position of each subject in the resulting coordinate system (score plot) was determined by his or her own profile of variables in relation to the other subjects’ profiles. The contribution of each variable to the positioning of the subjects was calculated, and the resulting loading values (in a loading plot) were used to interpret patterns (PCA), relationships (OPLS-DA), and co-dependencies (O2PLS) in the data. In other words, for the PCA, systematic data variability was extracted and the information was summarized in principal components and then the relationship between this systematic variability and sample collection time point (pre and post supplementation) was investigated in OPLS-DA. Finally, in O2PLS, this pre *vs.* post relationship between subsets of variables was further scrutinized to uncover co-dependencies among groups of variables. Model validity was assessed by the amount of systematic variation among the variables that were captured by the model (R2X(cum) and (R2Y(cum)), the predictive ability of the model (Q2(cum)), and p-values calculated by ANOVA based on the cross-validated score vectors (CV-ANOVA) [35]. All data were scaled to unit variance and mean-centered before modelling in order to prevent biased results due to the wide range of numerical values displayed between the different variables.

## Results

Out of the 13 subjects who were enrolled, 1 subject dropped out after the first day of the study due to gastrointestinal symptoms (diarrhea) that may or may not have been related to the intervention. The 12 remaining subjects included in the study completed the entire regimen and none reported adverse effects or problems in consuming the quantities of fish oil administered. The subjects’ mean (+/- SD) baseline mol % composition of key FA was: PC EPA 0.9% (0.5), PC DHA 3.4% (1), PC ARA 9.8% (2.1), PC EPA/ARA ratio 0.2 (0.04), PE EPA 1.0% (0.6), PE DHA 6.5% (1.5), PE ARA 27% (1.7), and PE EPA/ARA ratio 0.04 (0.02), as shown in Table 1. The mean (+/- SD) fold-increase in mol % composition of key FA was: PC EPA 6.7 (5.1), PC DHA 0.88 (0.59), PE EPA 5.5 (3.7), PE DHA 0.47 (0.45). 

Five out of the 12 subjects provided dietary records. These were analyzed by Nutrihand software and the averages of estimated intakes of macronutrients, some of the key micronutrients related to antioxidant function (i.e. vitamin C, vitamin E, vitamin A), and FA (i.e. ALA, LA, ARA, EPA and DHA as well as totals for saturated, monounsaturated, and polyunsaturated FA) were assessed by paired t-test for diet records provided before vs. after the intervention. The results are reported in Table **S1**. There were no significant changes in diet except the estimated intake of 18:2n6 was higher pre vs. post intervention (mean ± SD: 3.65 ± 2.27 g/d pre vs. 0.90 ± 1.56 g/d post; p = 0.03) in these 5 subjects. 

 Variables that were excluded from analysis because they either had >33% missing variables or were not detected (i.e. were below the limit of quantitation) are listed in Table **S2**. 

### Multivariate Analysis

#### PCA

Data examined by PCA produced strong models with regard to R2X(cum) for the complete lipidomic profile, oxylipins, and lipoprotein profiles (Figures **S2, S3**, and **S4**), as well as for each lipid class (data not shown). A summary of model assessment parameters (for all models included in the study) is found in Table **S3**. Lipidomic (Figure S2) and oxylipin (Figure S3) profiles were clearly separated by time point, with pre data for all subjects grouping together, and post data for all subjects grouping together, except for two individuals (202 and 220) with different lipidomic profiles compared to the rest of the subjects. Gender, age, weight, BMI, and weight adjusted EPA and DHA dose, displayed loading values close to zero, and were therefore not regarded as influential variables on the time point separation in either of the models obtained for complete lipidomic or oxylipin profiles. On the other hand, the lipoprotein profiles (Figure S4) were not strongly separated by time point, but instead by gender with sufficient loading values displayed for gender to drive this separation. 

However, the predictive power of these PCA analyses was in general weak (0.13 < Q2(cum) < 0.56). Two different approaches were taken to better understand this observation: further multivariate examination by OPLS-DA and visualization of individual responses followed by univariate statistical analysis. The OPLS-DA analysis was performed in order to understand which changes were consistent among all of the subjects, whereas the visualization and univariate analysis was performed in order to understand the changes that were inconsistent among subjects.

#### OPLS-DA and O2PLS Analysis

**Figure 1:**
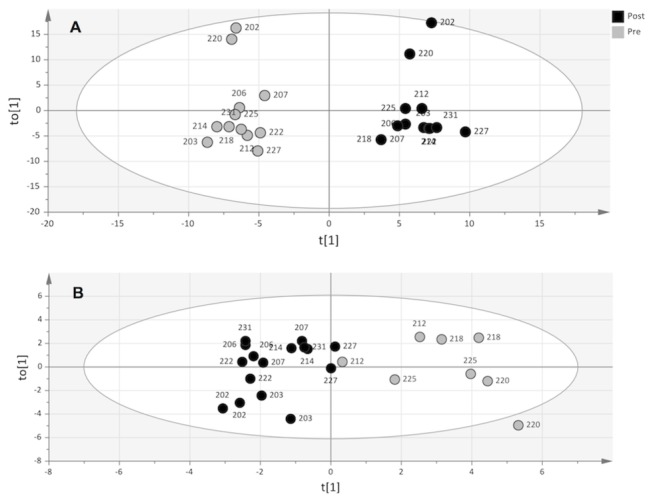
Lipidomic (A) and lipoprotein (B) profiles summarized as scores (t(1) and to(1)) calculated by OPLS-DA. All participants displayed altered lipidomic profiles pre (grey circles) vs. post (black circles) supplementation (A). Furthermore, the subjects displayed gender-specific lipoprotein profiles, female (black circles) and male (grey circles), both before and after intervention (B).

*Lipidomic Profiles:* Statistical examination of the total lipidomic data with OPLS-DA, revealed a significant difference (p = 0.00007) between lipidomic profiles pre and post administration with ω3 (Figure **1a**). The subjects were divided into two groups (pre and post) along the first component (x-axis), capturing most of the variability in the data set related to the time point of sample collection (14%). EPA and DHA incorporated into PC, CE, PE, TG, LY, total ω3 FA in PE, PC, CE and PC, as well as CYP-products of EPA and DHA were among the most influential species of the post-profile (Table **S4**). 

**Figure 2:**
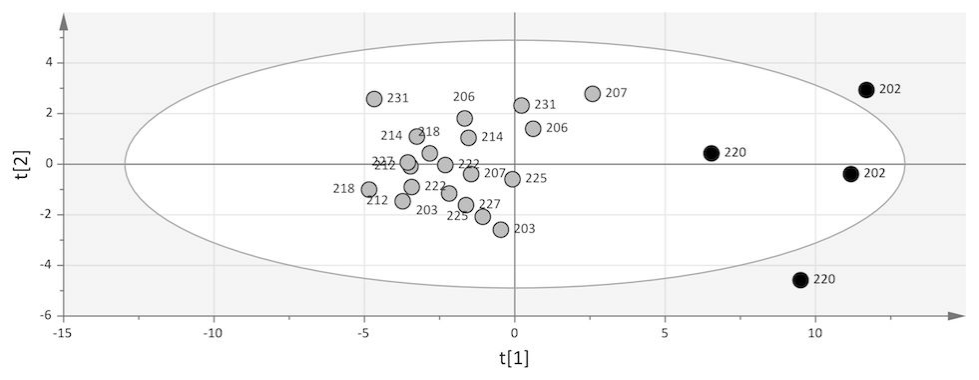
PCA score plot of the 35 variables most influential to the orthogonal component in Figure 1a. There was a pattern among the data separating subject 202 and 220 (black circles) from the rest of the subjects (grey circles).

Interestingly, this OPLS-DA analysis highlighted that two subjects, 202 and 220, separated from the rest of the subjects along the second component (y-axis), which captured most of the orthogonal variability, unrelated to time point (16%). The reason for this separation was not revealed in any of the PCA models for each lipid class, oxylipins, or lipoproteins. Therefore, identification of characteristics in common to both 202 and 220 was accomplished by detecting influential species to the orthogonal component (y-axis) in Figure 1a in order to interrogate the drivers separating subjects 202 and 220 from the rest of the group. A selection of the 35 most influential species to the orthogonal component revealed the substantial contribution of monounsaturated FA, n7 FA, and n9 FA, and other indicators of high de novo lipid synthesis (Table **S5**). A PCA model of these selected variables showed a clear separation of subjects 202 and 220 from the rest of the group (Figure **2**). Hence, the deviating behavior of subject 202 and 220 compared to the rest of the participants in the study was due to their increased markers of de novo lipid synthesis. Interestingly, neither the lipoprotein profile alone, nor the lipoprotein markers of de novo lipid synthesis displayed a difference that was specific to subjects 202 and 220. Hence, the variability in the lipidomic profiles not related to time point (pre vs. post) revealed by the orthogonal component in Figure 1a, which was responsible for the exclusive behavior of subjects 202 and 220, was partly offset by the gender aspect of the lipoprotein profile (Figure S4). 

The statistical interpretations of lipid changes across the subject population were extended by univariate analysis. Paired t-tests of all of the variables pre vs. post supplementation showed that PC, CE, PE, TG, and LY EPA and DHA had the largest and most consistent changes in response to intervention, with magnitude of change on the order of 2-, to 6-fold (Table **2**). EPA and DHA diols and one DHA epoxide, plus 5-oxo-ETE also increased significantly, whereas the ω6 FA LA and its elongation and desaturation intermediates 18:3n6, 20:3n6, 22:4n6, and 22:5n6 in PC, PE and CE decreased. Table **S6** shows additional metabolites that were increased or decreased post supplementation significant at an α≤0.005, which is not as stringent as the multiple comparison adjusted α of ≤ 0.0002. Notably, EPA LOX metabolites 12-HEPE, 15-HEPE and 5-HEPE increased; the EPA elongation product 22:5n3 increased in PC, CE, and FFA; several additional DHA diols increased; LA and ALA LOX metabolites 9-HODE and 9-oxo-ODE, and 13-HOTrE and 9-HOTrE respectively, decreased; total TG as well as the number of VLDL and chylomicron particles and their TG content decreased; the auto-oxidation markers EKODE decreased; and PC20:3n9, a marker of essential fatty acid deficiency, also decreased.

**Table 2:** Variables significantly different by paired t-test pre vs. post intervention at α ≤ 0.0002 (adjusted for multiple comparisons).

**Metabolite**	**Mean Pre**	**SD Pre**	**Mean Post**	**SD Post**	***P*-value**
Metabolites that increased post supplementation					
PC22:6n3 (nmol/g)	127	34.7	215	36.6	< 0.0001
PC20:5n3 (nmol/g)	32.8	19.9	182.2	53.9	< 0.0001
CE20:5n3 (nmol/g)	27.0	17.8	152.1	59.3	< 0.0001
TG22:6n3 (nmol/g)	13.5	6.63	69.6	26.5	< 0.0001
TG22:5n3 (nmol/g)	8.18	2.50	21.8	9.85	0.0001
TG20:5n3 (nmol/g)	4.87	3.53	50.4	21.0	< 0.0001
PE20:5n3 (nmol/g)	4.44	2.34	22.8	9.25	< 0.0001
LY20:5n3 (nmol/g)	0.588	0.413	3.28	1.42	< 0.0001
17,18-DiHETE (nmol/L)	0.476	0.155	2.68	1.35	< 0.0001
5-oxo-ETE (nmol/L)	0.458	0.122	3.46	1.91	< 0.0001
16,17-DiHDPE (nmol/L)	0.245	0.075	0.492	0.170	0.0002
10(11)-EpDPE (nmol/L)	0.162	0.094	0.548	0.271	< 0.0001
8,9-DiHETE (nmol/L)	0.069	0.031	0.457	0.223	< 0.0001
14,15-DiHETE (nmol/L)	0.060	0.027	0.413	0.221	< 0.0001
16(17)-EpDPE (nmol/L)	0.056	0.046	0.222	0.121	0.0001
11,12-DiHETE (nmol/L)	0.040	0.022	0.243	0.115	< 0.0001
Metabolites that decreased post supplementation					
PC18:2n6 (nmol/g)	958.54	212.13	779.99	206.65	0.0002
CE18:3n6 (nmol/g)	25.6	12.6	13.2	7.28	0.0001
PC22:4n6 (nmol/g)	12.0	2.54	6.95	2.15	< 0.0001
PE20:3n6 (nmol/g)	8.59	2.91	5.32	2.45	< 0.0001
PC22:5n6 (nmol/g)	8.49	3.43	3.86	1.52	< 0.0001

**Figure 3:**
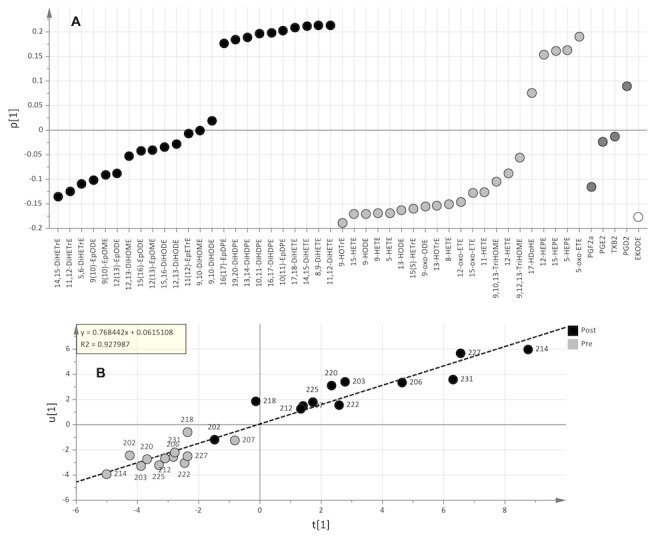
Contribution of each oxylipin to the pre (below origin) and post (above origin) profiles calculated by OPLS-DA (A). The CYP pathway (black) was the most responsive pathway, followed by the LOX pathway (light grey), and COX pathway (dark grey). Correlation between the oxylipin profiles and DHA and EPA incorporated in different lipid classes (B).

*Oxylipin Profiles:* OPLS-DA was also used to further evaluate the specific responses in oxylipin profiles. A significant difference (p = 0.000002) was detected between oxylipin profiles pre and post supplementation (Figure **S5**). CYP-products, especially those derived from DHA and EPA, as well as a subset of the total possible LOX products of EPA were increased post supplementation (Figure **3a**). Metabolites were grouped according to pathway and shown in a loading plot. Within each pathway, the metabolites are ordered by increasing influence to the differences associated with the post intervention profile. Hence, metabolites below the origin dominated the pre-intervention profile and vice versa. The larger the absolute value of the metabolite in the OPLS-DA analysis, the more influential in either the pre-intervention profile (negative values) or post-intervention profile (positive values) the metabolite was. The CYP pathway contained the most influential metabolites of the post profile, shown far to the left in Figure 3a, followed by LOX and COX pathway metabolites. 

Computational methods were then applied to interrogate the specific effects of each ω3 FA. The co-dependency between oxylipin profiles and DHA and EPA incorporated into different lipid classes was investigated in more depth by O2PLS (Figure **3b**). The components summarizing most of the oxylipin profile variability (x-axis) and DHA and EPA profile variability (y-axis) were plotted against each other. There was a strong overall correlation (r^2^=0.93), as well as individual correlation to each lipid class (Table **S7**). Hence, there was a joint variation among the oxylipins and DHA and EPA, which covered 30% of the total oxylipin and 66% of the total DHA and EPA variation. The major oxylipins correlating to EPA and DHA were CYP products of EPA and DHA (DiHETEs, and EpDPEs and DiHDPEs respectively) (Table **S8**), in accordance with the most influential species responsible for the statistically significant changes in the lipidomic profile pre vs. post supplementation. By extracting the 20% of the systematic variation in the oxylipin profile that was not related to EPA and DHA variation, LOX-products of LA (HODEs), ALA (HOTrEs), and ARA (HETEs) were detected to have little or no correlation with EPA and DHA (Table **S9**), meaning these metabolites were not responsive to the ω3 intervention.

*Lipoprotein Profiles:* The lipoprotein profiles of individuals prior to and after ω3 intervention were examined statistically using OPLS-DA. These analyses found a large and significant separation of individuals according to gender (Figure **1b**). Females showed higher values for HDL size, LDL size, large LDL particles, HDL cholesterol, large HDL particles, VLDL size, and medium VLDL particles, while males had higher total TG, total LDL particles, very small LDL particles, small LDL particles, medium small LDL particles, total VLDL and chylomicron particles, IDL particles, HDL particles, small VLDL particles, large VLDL and chylomicron particles, and small HDL particles. 

### Univariate Analysis

#### Individual Variation in ARA Metabolites

**Figure 4:**
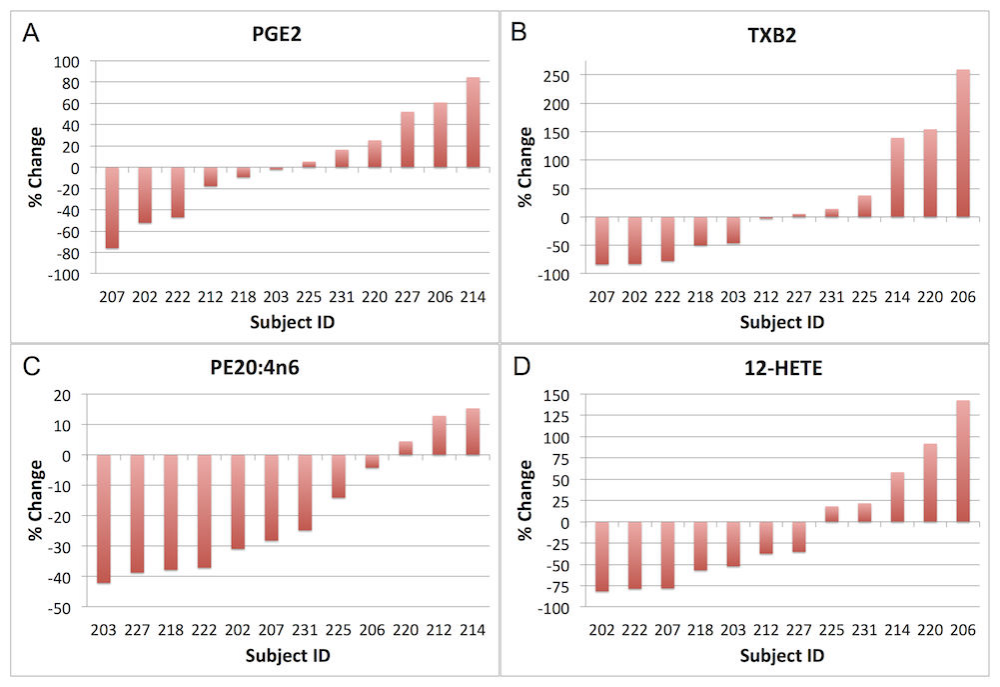
Bar graphs of each individual subject’s % change in the following arachidonic acid (ARA, 20:4n6) metabolites (subjects ordered by direction and magnitude of response on x axis): A) the cyclooxygenase (COX) metabolite prostaglandin E_2_ (PGE_2_); B) the COX metabolite thromboxane B_2_ (TXB_2_); C) ARA concentrations within the phosphatidylethanolamine (PE) lipid class; and D) the lipoxygenase metabolite 12-hidroxyeicosatetraenoic acid (12-HETE).

**Figure 5:**
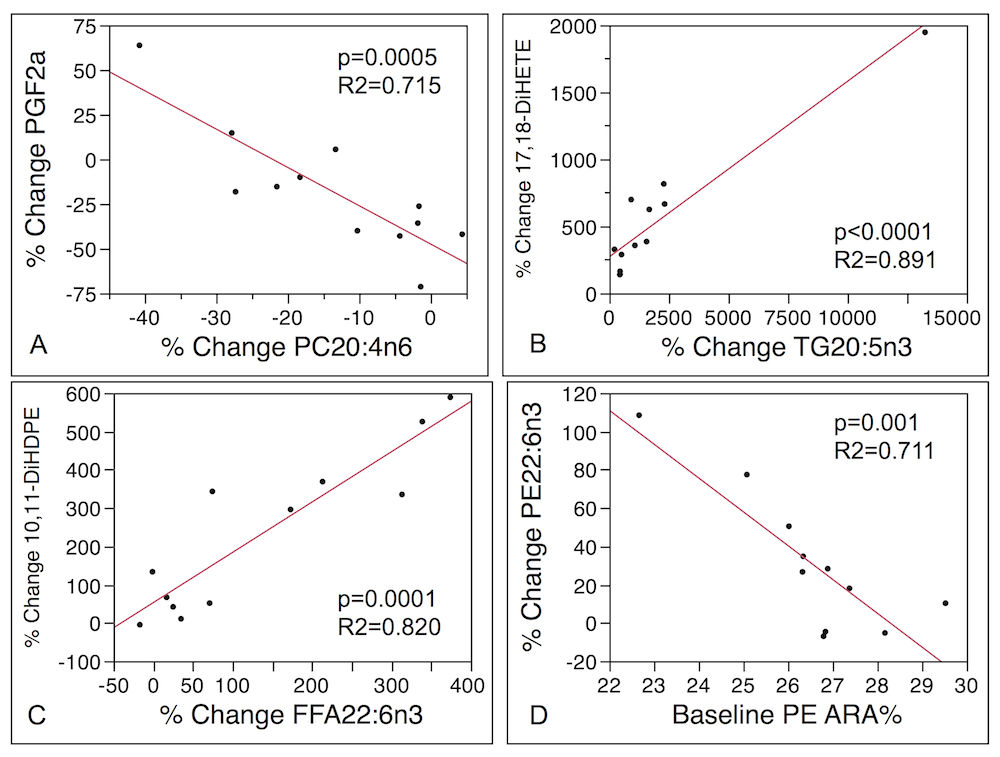
Correlations between: A) % change in arachidonic acid (ARA, 20:4n6) cyclooxygenase metabolite prostaglandin F_2α_ (PGF_2α_) and % change in phosphatidylcholine (PC) concentrations of ARA; B) % change in eicosapentaenoic acid (EPA, 20:5n3) soluble epoxide hydrolase (sEH) metabolite 17,18-dihydroxyeicosatrienoic acid (17,18-DiHETE) and % change in triglyceride (TG) concentrations of EPA; C) % change in docosahexaenoic acid (DHA, 22:6n3) sEH metabolite 10,11-dihydroxydocosapentaenoic acid (10,11-DiHDPE) and % change in free fatty acid (FFA) concentrations of DHA; D) % change in PE concentrations of DHA and baseline PE ARA %.

 Examining the responses of subjects as individuals was revealing. Several ARA derived eicosanoids known to be important in inflammatory disease processes including the COX-derived prostaglandin PGE_2_ and thromboxane TXB_2_, as well as the LOX-derived 12-hydroxyeicosatetraenoic acid (12-HETE) exemplify this variability. A bar graph of % change in PGE_2_ showed that in 6 out of 12 subjects (202, 203, 207, 212, 218, 222) PGE_2_ decreased, and in 6 out of 12 subjects (206, 214, 220, 225, 227, 231) PGE_2_ increased in response to the intervention (Figure **4a**). The magnitude of change ranged from a 76% decrease in subject 207 to an 85% increase in subject 214. Percentage change in PGE_2_ was not correlated with % change or baseline values of ARA. However, % change in ARA across several lipid classes was positively correlated with % changes in PGD_2_ and the HETEs (5-, 8-, 9-, and 12-HETE) (Table **3**). Percentage change in PC ARA was strongly negatively correlated with % change in PGF_2α_ such that the greater the decrease in the PC ARA content, the higher the increase in PGF_2α_ (Figure **5a**). The response in PGF_2α_ was also diverse across the 12 subjects, from 10-70% decreases in 9 of the subjects to 5-60% increases in 3 of the subjects (Figure **S6**). 

**Table 3:** Correlations between % change in arachidonic acid (ARA, 20:4n6), eicosapentaenoic acid (EPA, 20:5n3) and docosahexaenoic acid (DHA, 22:6n3), their oxylipin products, and baseline values at p ≤ 0.05.

**Variable X**	**Variable Y**	**Rsquare**	**P-value**
% Change CE20:4n6	% Change PGF_2α_	-0.501	0.015
% Change PE20:4n6	% Change PGD_2_	0.378	0.033
% Change PC20:4n6	% Change PGF_2α_	-0.715	0.001
% Change PE20:4n6	% Change TXB_2_	0.469	0.014
% Change PE20:4n6	% Change 12-HEPE	0.402	0.027
% Change PE20:4n6	% Change CE22:6n3	0.506	0.021
% Change FFA20:4n6	% Change 5-HETE	0.561	0.005
% Change PE20:4n6	% Change 8-HETE	0.340	0.047
% Change TG20:4n6	% Change 9-HETE	0.522	0.008
% Change PE20:4n6	% Change 12-HETE	0.427	0.021
% Change FFA20:4n6	% Change 15(S)-HETrE	0.383	0.032
% Change FFA20:4n6	% Change 5-HEPE	0.758	0.0002
Body Weight (kg)	% Change 8,9-DiHETE	-0.446	0.018
Body Weight (kg)	% Change 17,18-DiHETE	-0.521	0.012^a^
Body Weight (kg)	% Change 10,11-DiHDPE	-0.477	0.013
Body Weight (kg)	% Change 15-HEPE	-0.408	0.025
Body Weight (kg)	% Change 17-HDoHE	-0.353	0.042
Body Weight (kg)	% Change Medium VLDL Particles	0.394	0.039
Body Weight (kg)	% Change HDL Size (nm)	-0.326	0.052
Body Weight (kg)	% Change DG22:6n3	-0.525	0.018
Body Weight (kg)	% Change FFA18:3n6	-0.414	0.033
Body Weight (kg)	% Change FFA20:5n3	-0.519	0.008
Body Weight (kg)	% Change FFA22:6n3	-0.406	0.026
Body Weight (kg)	% Change PE22:0	0.476	0.027
Body Weight (kg)	% Change TG22:0	-0.484	0.025
Body Weight (kg)	Baseline PE EPA%	0.363	0.038
Body Weight (kg)	Baseline PE EPA/ARA	0.325	0.053
BMI	Baseline PC ARA%	0.431	0.020
BMI	Baseline PE EPA%	0.496	0.011
BMI	Baseline PE EPA/ARA	0.461	0.015
% Change FFA20:5n3	% Change 8,9-DiHETE	0.439	0.019
% Change FFA20:5n3	% Change 11,12-DiHETE	0.409	0.025
% Change DG20:5n3	% Change 14,15-DiHETE	0.691	0.003
% Change TG20:5n3	% Change 17,18-DiHETE	0.891	<0.0001^b^
% Change FFA20:5n3	% Change 17,18-DiHETE	0.344	0.045
% Change CE20:5n3	% Change 17,18-DiHETE	0.860	<0.0001^b^
% Change FFA20:5n3	% Change 5-HEPE	0.643	0.002
% Change CE20:5n3	% Change 15-HEPE	0.466	0.021
% Change FFA20:5n3	% Change 15-HEPE	0.666	0.001
% Change PC20:5n3	% Change 15-HEPE	0.452	0.017
% Change FFA22:6n3	% Change 10(11)-EpDPE	0.468	0.014
% Change FFA22:6n3	% Change 10,11-DiHDPE	0.820	0.0001
% Change FFA22:6n3	% Change 13,14-DiHDPE	0.488	0.011
% Change FFA22:6n3	% Change 16,17-DiHDPE	0.544	0.006
% Change TG22:6n3	% Change 19,20-DiHDPE	0.360	0.039
% Change FFA22:6n3	% Change 19,20-DiHDPE	0.432	0.020
% Change FFA22:6n3	% Change 17-HDoHE	0.690	0.001
% Change TG22:6n3	% Change 17-HDoHE	0.467	0.014
Baseline PC ARA%	% Change 5-HEPE	-0.350	0.043
Baseline PC ARA%	% Change 15-HEPE	-0.399	0.028
Baseline PE ARA%	% Change PE22:6n3	-0.711	0.001^c^
Baseline PC EPA%	% Change 5-HEPE	-0.335	0.049
Baseline PC EPA%	% Change 12-HEPE	-0.344	0.045
Baseline PC EPA%	% Change 15-HEPE	-0.412	0.024
Baseline Medium VLDL Particles	% Change DG22:6n3	0.416	0.044
Baseline LDL Size	% Change PC22:6n3	-0.372	0.035
Baseline HDL Size	% Change PC22:6n3	-0.327	0.052

^a^ Outlier removed: Subject 214

^b^ Subject 214 was considered a hyperresponder and was kept in the analysis, rather than being excluded as an outlier

^c^ Outlier removed: Subject 220

TXB_2_ also decreased in 5 subjects (202, 203, 207, 218, 222), stayed about the same in 2 subjects (212 and 227), and increased in 5 subjects (206, 214, 220, 225, 231) (Figure **4b**). The magnitude of change ranged from a decrease of 84% in subject 207 to an increase of 260% in subject 206. Percentage change in TXB_2_ was positively correlated with % change in PE ARA however, the p-value did not meet the Bonferroni-adjusted level of significance (p=0.01) and the R^2^ value was moderate (r^2^=0.47) (Table 3). The % change in PE ARA across individuals ranged from a 42% decrease in subject 203 to a 15% increase in subject 214 (Figure **4c**). The ARA LOX metabolite 12-HETE showed a similar pattern, with 12-HETE increasing in 5 subjects (206, 214, 220, 225, 231), and decreasing in 7 subjects (202, 203, 207, 212, 218, 222, 227) (Figure **4d**). The magnitude of change ranged from an 82% decrease in subject 202 to a 143% increase in subject 206. 

#### Individual Variation in EPA Metabolites

**Figure 6:**
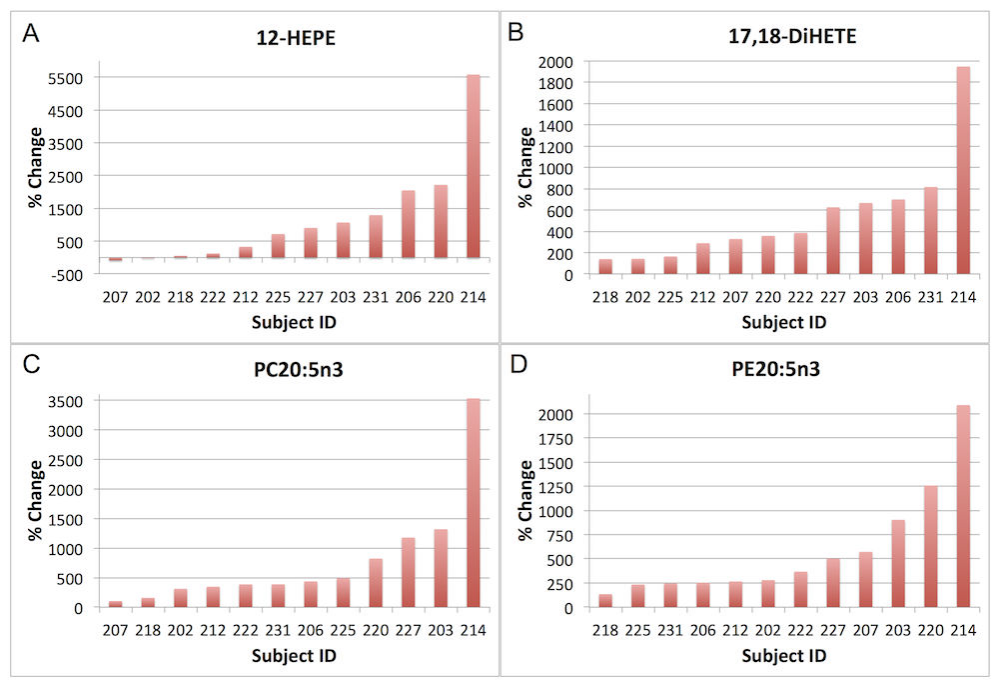
Bar graphs of each individual subject’s % change in the following eicosapentaenoic acid (EPA, 20:5n3) metabolites (subjects ordered by direction and magnitude of response on x axis): A) the lipoxygenase metabolite 12-hydroxyeicosapentaenoic acid (12-HEPE); B) the soluble epoxide hydrolase (sEH) metabolite 17,18-dihydroxyeicosatrienoic acid (17,18-DiHETE); C) phosphatidylcholine (PC) concentrations of EPA; and D) phosphatidylethanolamine (PE) concentrations of EPA.

The individual variability was equally evident in the changes in ω3 FA derived metabolites. The EPA LOX metabolite 12-hydroxyeicosapentaenoic acid (12-HEPE) displayed a large degree of variation from an 82% decrease in subject 207, to no change in subject 202, to increases in the range of 50-100% in subjects 222 and 218, to increases in the range of 300-1200% in subjects 225, 227, 203, 212, and 231, to increases in the range of 2000-5000% in subjects 220, 206, and 214 (Figure **6a**). Interestingly, three subjects (206, 214, and 220) who had high PGE_2_, TXB_2_, and 12-HETE also had high 12-HEPE concentrations post intervention. Baseline PC EPA % was negatively correlated with % change in 12-HEPE (Table 3), although this correlation did not meet the Bonferroni-adjusted p-value requirement for significance. 

The EPA diol 17,18-DiHETE increases ranged from 140% in subject 218 to 1947% in subject 214, an order of magnitude difference (Figure **6b**). This type of variation was observed across the EPA and DHA epoxides and diols (Figure S6). Percentage change in 17,18-DiHETE was highly correlated with % change in EPA in the TG (Figure **5b**) and CE lipid classes with R^2^ values of 0.89 and 0.86 respectively and p-values < 0.0002 (Table 3). Subject 214 was considered a hyper-responder rather than an outlier and was therefore kept in the analysis, but even when this subject was removed from the analysis, the correlations remained (TG: p=0.002, r^2^=0.66; CE: p=0.05; r^2^=0.4). EPA in the PC (Figure **6c**) and PE (Figure **6d**) lipid classes increased across all subjects however, to different degrees, with increases in PC EPA ranging from as low as 104% in subject 207 to as high as 3528% in subject 214. Subject 214 was a hyper-responder and had the highest increases in all 4 metabolites (PC and PE EPA, 12-HEPE and 17,18-DiHETE). 

#### Individual Variation in DHA Metabolites

**Figure 7:**
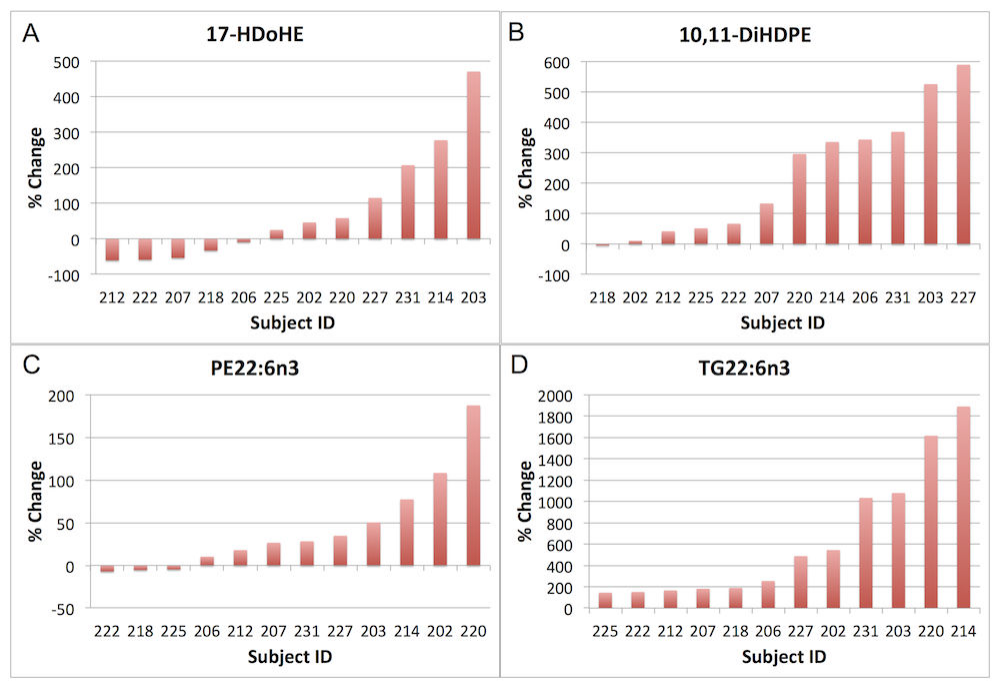
Bar graphs of each individual subject’s % change in the following docosahexaenoic acid (DHA, 22:6n3) metabolites (subjects ordered by direction and magnitude of response on x axis): A) the lipoxygenase metabolite 17-hydroxy docosahexaenoic acid (17-HDoHE); B) the soluble epoxide hydrolase (sEH) metabolite 10,11-dihydroxydocosapentaenoic acid (10,11-DiHDPE); C) phosphatidylethanolamine (PE) concentrations of DHA; and D) triglyceride (TG) concentrations of DHA.

Responsiveness in the DHA metabolites also varied across individual subjects. Percentage change in the DHA LOX metabolite 17-HDoHE ranged from a decrease of 60% in subject 212 to an increase of 470% in subject 203 (Figure **7a**). Percentage change in 17-HDoHE was positively correlated with % change in both FFA and TG DHA, as well as being negatively correlated with body weight (Table 3). The % change in DHA diol 10,11-DiHDPE ranged from essentially no change (-4% in subject 218) to an increase of 589% in subject 227 (Figure **7b**). 10,11-DiHDPE was positively correlated with DHA in the FFA lipid class (Figure **5c**) and negatively correlated with body weight (Table 3). Changes in DHA across lipid classes also varied, as exemplified by PE and TG. PE DHA ranged in % change from essentially no change (-4 to -6% in subjects 218, 222, and 225) to an increase of 187% in subject 220 (Figure **7c**), whereas TG DHA ranged in % change from about 200% in subjects 206, 207, 212, 218, 222, and 225 to 1890% in subject 214 (Figure **7d**). Baseline PE ARA% was negatively correlated with PE DHA (Table 3) such that the lower the starting PE ARA% the higher the increase in PE DHA (Figure **5d**). 

#### Age, Gender, Body Mass, and Baseline Effects

This was a pilot study not specifically designed and powered to adjust for covariates. However, for exploratory purposes, standard least squares linear regressions on the % change of all variables were run and adjusted for age, gender, body weight, and BMI, and those that reached p-values < 0.05 for the overall model were reported in Table **S10**. None of the p-values were below the Bonferroni-adjusted level of α < 0.0002 for the overall model. However, there were some notable effects including a strong influence of age, body weight, and BMI on the response in PC:18:0. Percentage change in PC18:0 was negatively correlated with age and body weight, and positively correlated with BMI. Other notable effects included the effects of age, gender and body weight on response in CE18:1n9, the effects of age and body weight on response in large HDL particles, and the effect of BMI on the response in TG22:0 and TG22:1n9. Notably, responses in neither the FA precursors (i.e. EPA, DHA, and ARA) nor their oxylipin products were influenced by age, gender, body weight or BMI. 

 There were several gender effects observed when response in the metabolites was assessed by t-test comparing % change in females vs. males (Table **S11**). Response to the treatment in elongation products varied between females and males. Notably, whereas TG22:0 increased by about 50% in females, it decreased by the same amount in males. Similarly, TG 20:1n9 and TG22:1n9 increased in females and decreased in males. There were also some differences in lipoprotein particle numbers and sizes, as expected given the multivariate analysis results. Large VLDL & chylomicron particles decreased more in males, whereas medium VLDL particles decreased in the females but increased in males. The response in the ARA, EPA, and DHA epoxides and diols was also different between genders. Females had much higher increases in all EPA and DHA diols, with lesser decreases in ARA diols. Females also had higher weight-adjusted EPA and DHA doses.

## Discussion

In this study comprehensive multi-platform lipidomic profiling of 12 healthy individuals pre vs. post intervention with a proscribed dose of fish oil enriched in ω3 FA was performed in order to understand both the universal aspects of response to these lipids in healthy people and the variation in response among individuals. Both multivariate and univariate statistical approaches were applied to determine the consistent and variable aspects of response among these subjects in FA, oxylipins, and lipoproteins. 

We found a decrease of about 24% in plasma TG (p=0.0005), which is in agreement with previous studies and meta-analyses showing approximately 25–30% decreases in TG in healthy subjects in response to 3-4 g/d EPA + DHA [36,37]. VLDL and chylomicron particles also decreased by about 32% (p=0.005). Lipoprotein profiles were more indicative of gender differences rather than response to intervention. Female subjects had lipoprotein profiles that were more consistent with traditional markers of lower risk for heart disease (i.e. more and larger HDL, larger LDL) whereas male subjects had lipoprotein profiles that are associated with classic indicators of higher risk (i.e. more TG and LDL, more small LDL and small HDL particles). These findings are in agreement with previous findings of higher large HDL, and lower small and medium LDL, large and medium VLDL, and small HDL in women than in men [38]. In female subjects, medium VLDL decreased whereas in male subjects medium VLDL increased and there were greater decreases in VLDL and chylomicron particles. Previous studies have shown that ω3 shift VLDL toward smaller particles in men [39]. A previous study found that HDL decreased in women whereas TG decreased in men, however, this was after a much higher dose of EPA (4.5 g/d) and slightly higher dose of DHA (1.9 g/d) than in this study [40]. A recent study supplementing overweight subjects with almost exactly the same fish oil from Ocean Nutrition with the same amount of EPA but slightly lower DHA for the same duration (6 weeks) found that TG decreased by 11-25%, but that there were no effects on LDL, HDL, ApoB, insulin, or CRP when they examined men and women together [37]. However, they found that the intervention increased HDL in women, and they also found that apoE genotype was associated with fasting glucose, insulin, and CRP responses [37]. Thus it is likely that genetic differences in addition to gender play a role in the differential responses to ω3 FA. 

Multivariate analyses created strong models of pre vs. post differences in FA and oxylipin profiles, with plasma levels of EPA and DHA incorporated into different lipid classes and CYP products of EPA and DHA (DiHETEs, and EpDPEs and DiHDPEs respectively) increasing in all subjects. Some elongation and desaturation metabolites of ω6 FA decreased, confirming ω3 outcompeted the ω6 in the desaturation and elongation pathways, as has been observed previously [41]. The relative proportion of phospholipid EPA increased on average 6–7-fold, and phospholipid DHA 0.5–1-fold. This is in agreement with previous studies, which showed similar increases in phospholipid EPA and DHA [20,22,42]. Delta-5 desaturase (FADS1) and delta-6 desaturase (FADS2) polymorphisms have been shown to be highly influential in plasma, red blood cell, and milk EPA and DHA composition [43-47], and may have played a role in the divergent EPA and DHA levels among subjects in this study. Consistent increases in the EPA and DHA CYP products (EPA diols and DHA epoxides and diols) have been observed previously in response to long-chain ω3 supplementation in patients [21,22] and in healthy controls [20,22,42]. These metabolites (DHETEs, EpDPEs, DHDPEs) may be particularly informative because of their apparent dose-response relationship, and could be useful as markers of ω3 FA intake and responsiveness. Decreases in ARA epoxides and diols were not found to be statistically significant after correction for multiple comparisons in this study. 

The inter-individual variation in all metabolites in response to the ω3 FA intervention was explicitly examined, and was surprisingly high. In half of the subjects, not grouped according to gender or bodyweight, ARA-derived eicosanoids PGE_2_, TXB_2_, and 12-HETE increased post intervention whereas in the other half, the expected decrease in these metabolites was observed. PGF_2α_ decreased in 9 but increased in 3 subjects. Previous studies (in vitro, animal, and human) have found fish oil and ω3 FA reduce ARA-derived eicosanoids [14-18,48-51]. A recent study supplementing healthy men with similar EPA and DHA doses as in this study found increased TXB_2_ and PGF_2α_ and no change in 12-HETE but highlighted high inter-individual variability in response to the fish oil [52]. These authors also found that 12-HETE decreased in 2 participants but increased in 8 others [52]. 

There were no correlations between changes in PGE_2_ and either baseline ARA or % change in ARA. Percentage change in ARA was, however, correlated with % change in PGD_2_, TXB_2_, PGF_2α_ and the HETEs. The changes in ARA were not universal across subjects, with % change in, for instance, PE ARA ranging from a 42% decrease to a 15% increase. The dietary data from the 5 subjects who supplied adequate diet records did find modest changes in estimated intakes of LA, and these could be related to some of the observed differences in ARA concentration. However, given that ω3 FA inhibit LA conversion to ARA, as confirmed by the observed reduction in the intermediates 18:3n6 and 20:3n6, it is unlikely that dietary LA conversion to ARA played a significant role in the variability in ARA among subjects. Nonetheless, future studies will need to more adequately control for the intake of LA and ARA in the diet to completely account for diet as a potential source of ARA, which could affect the overall effect of the ω3 intervention. It is possible that dietary records will not be sufficiently accurate estimates of dietary FA intakes and that alternative methods for controlling dietary FA composition will be needed.

Whether the observed increases and decreases in plasma levels of oxylipins correlate with actual disease outcomes or are indicative of overall systemic inflammation is unknown. In general, outcome studies have not yet related the abundance of these important lipid mediators to either health or specific diseases. Therefore it is unclear whether the changes in concentration observed in this study – as much as a 6-fold decrease in TXB_2_ in subject 207, or a 2.5-fold increase in TXB_2_ in subject 220, for example – are indicative of deviations from normal that accurately predict an increase or decrease in disease risk. Previous studies have found coefficients of variation (CVs) in PGE_2_ on the order of 251% prior to ω3 intervention and 156% post intervention in asthmatics [22], whereas the CVs for PGE_2_ in this study were 36% and 28% respectively. Another study evaluating the human serum metabolome found a CV of 76% for PGE_2_ among 70 healthy subjects [53]. Thus, the variability in PGE_2_ among the healthy subjects in this study was lower than in asthmatic subjects and in a larger cohort of healthy subjects. Much still needs to be learned about the fluctuations over time in these metabolites among healthy individuals and in individuals with various disease states in order to understand the significance of observed changes in response to ω3 FA.

Even among the metabolites that ubiquitously increased in all subjects, there was a high degree of inter-individual variation in the magnitude of change. For example, EPA in PC and PE ranged in its increase from 2-fold to 36-fold among subjects. PE DHA ranged from no change to a 200% increase. TG DHA ranged from 200% to 2000% increase. This variability points to fundamental differences in metabolism that may be anywhere along the chain of events from intestinal absorption to incorporation into phospholipids to lipoprotein synthesis and turnover. Previous studies investigating EPA and DHA incorporation into serum or plasma lipid pools have shown increases in the range of 4-fold to 8-fold at similar supplementation doses as in the present study [54,55]. However, this study, as do most studies, reported total phospholipid FA mol % composition rather than quantitative concentrations within specific phospholipids or other lipid classes as we did in the present study. Much as cholesterol levels are diagnostic as absolute concentrations (mg/dL), larger studies are needed to determine the range of normal concentrations of the ω3 FA across the lipid classes and their metabolites across healthy individuals, and the deviations in the absolute concentrations that are associated with different disease conditions and diets. 

There were no significant correlations between baseline or % change in EPA, DHA, or ARA and age, gender, body weight, or BMI despite the fact that female subjects received higher effective doses of EPA and DHA (i.e. weight-adjusted doses). Similarly, there were no effects of age, gender, body weight, or BMI on % change in ARA, EPA, and DHA or their oxylipin products. However, there were some associations between precursor FA within specific lipid pools and several downstream oxylipin metabolites. Baseline PC EPA was negatively correlated with % change in 12-HEPE such that the lower the starting EPA concentration, the higher the increase in 12-HEPE. The EPA diol 17,18-DiHETE was strongly positively correlated with % change in EPA in the TG and CE lipid classes, but not the phospholipids. This metabolite appears to be a consistent marker of dose-dependent EPA supplementation. The lower the baseline PE ARA the higher the increase in PE DHA. This suggests that some individuals have high constitutive ARA in their PE that does not get replaced by DHA, whereas in other individuals there is a higher rate of replacement of ARA with DHA in this particular phospholipid class. Percentage change in the DHA LOX metabolite 17-HDoHE was positively correlated with % change in TG and FFA DHA, and the DHA diol 10,11-DiHDPE was also positively correlated with % change in FFA DHA. These data highlight high individual variation in the extent of incorporation of EPA and DHA into different plasma lipid pools and in the subsequent production of downstream oxylipin metabolites from these different pools. Further studies are needed to better understand how EPA and DHA and their metabolites are differentially metabolized in different dietary and genetic backgrounds, and the implications of these differences in the various outcomes related to ω3 interventions. 

This was an exploratory/pilot study with 12 subjects, which was not blinded and did not have a control arm, all of which are potential limitations of the study. The current study was not powered specifically to determine age, gender, body weight, or BMI effects, thus our observations in this area are exploratory in nature and highlight the need for further studies. This study also did not adequately control for diet and exercise/physical activity. We did not design this study explicitly to determine clinically relevant health outcomes related to ω3 intervention. Instead, our aim was to determine whether the comprehensive profiling of lipid metabolism to assess response to ω3 FA could be a useful approach for assessing individual responsiveness and to identify specific metabolic phenotypes. We have demonstrated that a comprehensive lipidomic approach does indeed reveal those aspects of responsiveness to ω3 FA that are consistent across healthy individuals and those that are variable. Future studies will need to determine the causes of the high variability in certain aspects of metabolism, and the implications of this variability on health outcomes so that the goal of a more personalized approach can be realized.

## Conclusions

Current intakes of ω3 FA in the U.S. are about 1.7 g/d out of a total of 19 g/d of polyunsaturated FA including both ω6 and ω3, with most of that being in the form of the 18C precursor ALA [6]. Intakes of the long-chain EPA and DHA are only about 130 mg/d [6]. This represents a ratio of ω6:ω3 of 10:1, whereas estimates of historic intakes are closer to 1:1 [56]. Thus ω3 FA are generally deficient in Western diets and these dietary deficiencies in ω3 FA may be contributing to the preponderance of inflammatory diseases in the U.S. and elsewhere. However, there is a lack of consensus about whether and how to establish dietary recommendations for the intake of ω3 due to conflicting findings in both observational and intervention studies. A recent comprehensive update from an expert panel concluded that a key knowledge gap in understanding the effects of ω3 FA in modifying disease risk is the ability to identify those groups of individuals for whom they are beneficial and those for whom they are not beneficial, as well as the effective dosages and formulations [57]. In the present study we have shown that a comprehensive multi-platform lipidomic approach, simultaneously measuring FA within different lipid classes, oxylipins, and lipoprotein particle size and distribution, is able to identify key differences in response to ω3 intervention. Larger studies utilizing a similar approach should address variation among men and women with different genetic backgrounds, on different background diets, and on different formulations and dosages of ω3 FA to assess the effectiveness of ω3 interventions on modifying metabolic, inflammatory, and lipoprotein profiles toward a healthier phenotype.

## Supporting Information

Figure S1 **Fatty acid precursors and their oxylipin products.** The fatty acids linoleic acid (LA, 18:2n6), α-linolenic acid (ALA, 18:3n3), arachidonic acid (ARA, 20:4n6), dihomo-γ-linolenic acid (DGLA, 20:3n6), eicosatrienoic acid (ETA, 20:3n9), eicosapentaenoic acid (EPA, 20:5n3), and docosahexaenoic acid (DHA, 22:6n3) are precursors to a number of oxylipin products produced via the cyclooxygenase (COX), lipoxygenase (LOX), and cytochrome P 450 (CYP) enzymes. The oxylipin products of the COX pathway include prostaglandins (PGE_1_, PGD_1_, PGH_2_, PGF_2α_. PGE_2_, PGB_2_, PGD_2_, PGJ_2_, 15-deoxy-PGJ_2_, PGI_2_, 6-keto-PGF_1α_, PGE_3_, PGH_3_, and resolvin E1) and thromboxanes (TXA_2_, TXB_2_). The oxylipin products of the LOX pathway include hydroperoxyeicosatetraenoic acids (HpETEs) and dihydroxyeicosatetraenoic acid (DiHETE), (further converted to hydroxyeicosatetraenoic acids (HETEs)), hydroxyoctadecadienoic acids (HOTrEs), hydroxyeicosaptenaenoic acids (HEPEs), hydrox- ydocosahexaenoic acid (17-HDoHE), and leukotrienes (LTA_4_, LTB_4_, 20-OH-LTB_4_, 20-COOH-LTB_4_, 6-trans-LTB_4_, LTC_4_, LTD_4_, LTE_4_, LTB_3_, LTB_5_) as well as the hydroxyoctadienoic acids (HODEs), and trihydroxyoctamonoenoic acids (TriHOMEs). The products of the CYP hydroxy (OH) pathway include 20-HETE, and the products of the CYP epoxy pathway include the epoxyeicosatrienoic acids (EETs), epoxyoctadecadienoic acids (EpODEs), epoxyoctamonoe- noic acids (EpOMEs), epoxyeicosatetreaenoic acids (EpETEs), and epoxydocosapentaenoic acids (EpDPEs), as well as the downstream soluble epoxide hydrolase (sEH) metabolites dihydroxyoctamonoe- noic acids (DiHOMEs), dihydroxyeicosatrienoic acids (DiHETrEs), dihydroxyoctadecadienoic acids (DiHODEs), dihydroxyeicosatetrae- noic acids (DiHETEs), and dihydroxydocosapentaenoic acids (DiHDPEs). Each fatty acid precursor and its oxylipin products are colored the same: LA, orange; DGLA, yellow; ETA, dark blue; ALA, purple; EPA, green; DHA, red; and ARA, light blue.
Reprinted with kind permission from Springer Science+Business Media: Metabolomics, Serum oxylipin profiles in IgA nephropathy patients reflect kidney functional alterations, volume 8, 2012, 1102-1113, Angela M. Zivkovic, Jun Yang, Katrin Georgi, Christine Hegedus, Malin L. Nording, Aifric O’Sullivan, J. Bruce German, Ronald J. Hogg, Robert H. Weiss, Curt Bay, Bruce D. Hammock, Figure 1.
(DOCX)

Figure S2 **Score plot of lipidomic profiles calculated by PCA.** There was a pattern among the data separating pre (grey circles) and post (black circles) lipidomic profiles.
(DOCX)

Figure S3 **Score plot of oxylipin profiles calculated by PCA.** There was a pattern among the data separating pre (grey circles) and post (black circles) oxylipin profiles.
(DOCX)

Figure S4 **Score plot of lipoprotein profiles calculated by PCA.** There was a pattern among the data separating male (grey circles) and female (black circles) lipoprotein profiles.
(DOCX)

Figure S5 **Score plot of oxylipin profiles in each individual subject calculated by OPLS-DA.** There was a clear separation between pre (grey circles) and post (black circles) profiles in all of the participants.
(DOCX)

Figure S6 **Bar graphs of each individual subject’s % change in the following metabolites (subjects ordered by direction and magnitude of response on x axis): A) the arachidonic acid cyclooxygenase metabolite prostaglandin F_2α_ (PGF_2α_); B) the soluble epoxide hydrolase (sEH) eicosapentaenoic acid (EPA, 20:5n3) metabolite 8,9-dihydroxyeicosatrienoic acid (8,9-DiHETE); C) the sEH docosahexaenoic acid (DHA, 22:6n3) metabolite 19,20-dihydroxydocosapentaenoic acid (19,20-DiHDPE); and D) the cytochrome P450 DHA metabolite 10(11)-epoxydocosapentaenoic acid (10(11)-EpDPE).**
(DOCX)

Table S1 **Dietary record summaries pre vs. post intervention averaged across 5 of the 12 subjects (mean and SD), assessed by paired t-test.**
(DOCX)

Table S2 **Variables that were excluded from analysis because they either had >33% missing variables or were not detected (i.e. were below the limit of quantitation).**
(DOCX)

Table S3 **Model assessment parameters.**
(DOCX)

Table S4 **Individual contributions of each variable to the post profile (high loading values) and pre profile (low loading values).**
(DOCX)

Table S5 **The 35 most influential variables to the orthogonal component in the OPLS-DA model (Figure 1a) separating subjects 202 and 220 from the rest of the group.**
(DOCX)

Table S6 **Additional metabolites that increased or decreased post intervention at α ≤ 0.005.**
(DOCX)

Table S7 **Lipid-specific p-values in the O2PLS model correlating joint variation between oxylipins and EPA and DHA.**
(DOCX)

Table S8 **Variables with joint variation according to O2PLS-DA modeling (the higher loading value, the more influential the variable).**
(DOCX)

Table S9 **Variables among the oxylipins with unique variation (and little or no correlation with EPA and DHA) according to O2PLS-DA modeling (the lower the value, the more influential).**
(DOCX)

Table S10 **Results from standard lease squares linear regressions for each variable adjusted for age, gender, body weight, and BMI, with p-values ≤ 0.05.**
(DOCX)

Table S11 **Gender differences in response to treatment.** Mean and Standard Error (SE) for females vs. males compared by t-test.
(DOCX)
