# Supplementary material for: Individual Variation in Lipidomic Profiles of Healthy Subjects in Response to Omega-3 Fatty Acids
Source: PLoS One. 2013 Oct 24;8(10):e76575. doi: 10.1371/journal.pone.0076575 (PMC3811983; doi:10.1371/journal.pone.0076575)
Supplement: Figure S1 — Fatty acid precursors and their oxylipin products. The fatty acids linoleic acid (LA, 18:2n6), α-linolenic acid (ALA, 18:3n3), arachidonic acid (ARA, 20:4n6), dihomo-γ-linolenic acid (DGLA, 20:3n6), eicosatrienoic acid (ETA, 20:3n9), eicosapentaenoic acid (EPA, 20:5n3), and docosahexaenoic acid (DHA, 22:6n3) are precursors to a number of oxylipin products produced via the cyclooxygenase (COX), lipoxygenase (LOX), and cytochrome P 450 (CYP) enzymes. The oxylipin products of the COX pathway include prostaglandins (PGE1, PGD1, PGH2, PGF2α. PGE2, PGB2, PGD2, PGJ2, 15-deoxy-PGJ2, PGI2, 6-keto-PGF1α, PGE3, PGH3, and resolvin E1) and thromboxanes (TXA2, TXB2). The oxylipin products of the LOX pathway include hydroperoxyeicosatetraenoic acids (HpETEs) and dihydroxyeicosatetraenoic acid (DiHETE), (further converted to hydroxyeicosatetraenoic acids (HETEs)), hydroxyoctadecadienoic acids (HOTrEs), hydroxyeicosaptenaenoic acids (HEPEs), hydrox- ydocosahexaenoic acid (17-HDoHE), and leukotrienes (LTA4, LTB4, 20-OH-LTB4, 20-COOH-LTB4, 6-trans-LTB4, LTC4, LTD4, LTE4, LTB3, LTB5) as well as the hydroxyoctadienoic acids (HODEs), and trihydroxyoctamonoenoic acids (TriHOMEs). The products of the CYP hydroxy (OH) pathway include 20-HETE, and the products of the CYP epoxy pathway include the epoxyeicosatrienoic acids (EETs), epoxyoctadecadienoic acids (EpODEs), epoxyoctamonoe- noic acids (EpOMEs), epoxyeicosatetreaenoic acids (EpETEs), and epoxydocosapentaenoic acids (EpDPEs), as well as the downstream soluble epoxide hydrolase (sEH) metabolites dihydroxyoctamonoe- noic acids (DiHOMEs), dihydroxyeicosatrienoic acids (DiHETrEs), dihydroxyoctadecadienoic acids (DiHODEs), dihydroxyeicosatetrae- noic acids (DiHETEs), and dihydroxydocosapentaenoic acids (DiHDPEs). Each fatty acid precursor and its oxylipin products are colored the same: LA, orange; DGLA, yellow; ETA, dark blue; ALA, purple; EPA, green; DHA, red; and ARA, light blue. Reprinted with kind permission from Springer Science+Busine [file pone.0076575.s001.docx]

**
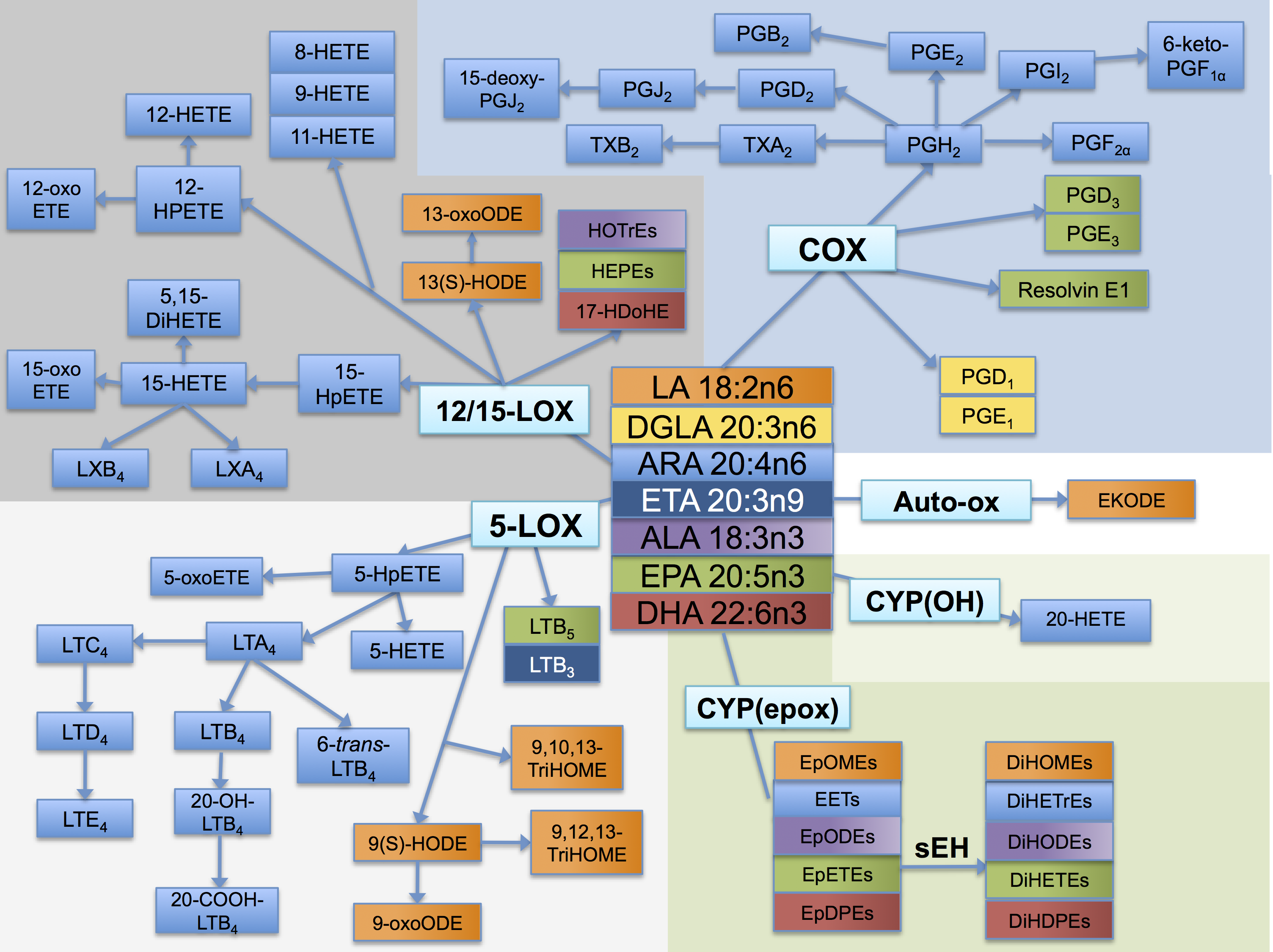
**

**Figure S1.** Fatty acid precursors and their oxylipin products. The fatty acids linoleic acid (LA, 18:2n6), α-linolenic acid (ALA, 18:3n3), arachidonic acid (ARA, 20:4n6), dihomo-γ-linolenic acid (DGLA, 20:3n6), eicosatrienoic acid (ETA, 20:3n9), eicosapentaenoic acid (EPA, 20:5n3), and docosahexaenoic acid (DHA, 22:6n3) are precursors to a number of oxylipin products produced via the cyclooxygenase (COX), lipoxygenase (LOX), and cytochrome P 450 (CYP) enzymes. The oxylipin products of the COX pathway include prostaglandins (PGE_1_, PGD_1_, PGH_2_, PGF_2α_. PGE_2_, PGB_2_, PGD_2_, PGJ_2_, 15-deoxy-PGJ_2_, PGI_2_, 6-keto-PGF_1α_, PGE_3_, PGH_3_, and resolvin E1) and thromboxanes (TXA_2_, TXB_2_). The oxylipin products of the LOX pathway include hydroperoxyeicosatetraenoic acids (HpETEs) and dihydroxyeicosatetraenoic acid (DiHETE), (further converted to hydroxyeicosatetraenoic acids (HETEs)), hydroxyoctadecadienoic acids (HOTrEs), hydroxyeicosaptenaenoic acids (HEPEs), hydrox- ydocosahexaenoic acid (17-HDoHE), and leukotrienes (LTA_4_, LTB_4_, 20-OH-LTB_4_, 20-COOH-LTB_4_, 6-trans-LTB_4_, LTC_4_, LTD_4_, LTE_4_, LTB_3_, LTB_5_) as well as the hydroxyoctadienoic acids (HODEs), and trihydroxyoctamonoenoic acids (TriHOMEs). The products of the CYP hydroxy (OH) pathway include 20-HETE, and the products of the CYP epoxy pathway include the epoxyeicosatrienoic acids (EETs), epoxyoctadecadienoic acids (EpODEs), epoxyoctamonoe- noic acids (EpOMEs), epoxyeicosatetreaenoic acids (EpETEs), and epoxydocosapentaenoic acids (EpDPEs), as well as the downstream soluble epoxide hydrolase (sEH) metabolites dihydroxyoctamonoe- noic acids (DiHOMEs), dihydroxyeicosatrienoic acids (DiHETrEs), dihydroxyoctadecadienoic acids (DiHODEs), dihydroxyeicosatetrae- noic acids (DiHETEs), and dihydroxydocosapentaenoic acids (DiHDPEs). Each fatty acid precursor and its oxylipin products are colored the same: LA, orange; DGLA, yellow; ETA, dark blue; ALA, purple; EPA, green; DHA, red; and ARA, light blue.

Reprinted with kind permission from Springer Science+Business Media: *Metabolomics*, Serum oxylipin profiles in IgA nephropathy patients reflect kidney functional alterations, volume 8, 2012, 1102-1113, Angela M. Zivkovic, Jun Yang, Katrin Georgi, Christine Hegedus, Malin L. Nording, Aifric O’Sullivan, J. Bruce German, Ronald J. Hogg, Robert H. Weiss, Curt Bay, Bruce D. Hammock, figure 1.
