# Supplementary material for: Individual Variation in Lipidomic Profiles of Healthy Subjects in Response to Omega-3 Fatty Acids
Source: PLoS One. 2013 Oct 24;8(10):e76575. doi: 10.1371/journal.pone.0076575 (PMC3811983; doi:10.1371/journal.pone.0076575)
Supplement: Figure S3 — Score plot of oxylipin profiles calculated by PCA. There was a pattern among the data separating pre (grey circles) and post (black circles) oxylipin profiles. (DOCX) [file pone.0076575.s003.docx]

**Figure S3.** Score plot of oxylipin profiles calculated by PCA. There was a pattern among the data separating pre (grey circles) and post (black circles) oxylipin profiles.
