# Supplementary material for: Individual Variation in Lipidomic Profiles of Healthy Subjects in Response to Omega-3 Fatty Acids
Source: PLoS One. 2013 Oct 24;8(10):e76575. doi: 10.1371/journal.pone.0076575 (PMC3811983; doi:10.1371/journal.pone.0076575)
Supplement: Figure S5 — Score plot of oxylipin profiles in each individual subject calculated by OPLS-DA. There was a clear separation between pre (grey circles) and post (black circles) profiles in all of the participants. (DOCX) [file pone.0076575.s005.docx]

**Figure S5.** Score plot of oxylipin profiles in each individual subject calculated by OPLS-DA. There was a clear separation between pre (grey circles) and post (black circles) profiles in all of the participants.
