# Supplementary material for: Individual Variation in Lipidomic Profiles of Healthy Subjects in Response to Omega-3 Fatty Acids
Source: PLoS One. 2013 Oct 24;8(10):e76575. doi: 10.1371/journal.pone.0076575 (PMC3811983; doi:10.1371/journal.pone.0076575)
Supplement: Figure S6 — Bar graphs of each individual subject’s % change in the following metabolites (subjects ordered by direction and magnitude of response on x axis): A) the arachidonic acid cyclooxygenase metabolite prostaglandin F2α (PGF2α); B) the soluble epoxide hydrolase (sEH) eicosapentaenoic acid (EPA, 20:5n3) metabolite 8,9-dihydroxyeicosatrienoic acid (8,9-DiHETE); C) the sEH docosahexaenoic acid (DHA, 22:6n3) metabolite 19,20-dihydroxydocosapentaenoic acid (19,20-DiHDPE); and D) the cytochrome P450 DHA metabolite 10(11)-epoxydocosapentaenoic acid (10(11)-EpDPE). (DOCX) [file pone.0076575.s006.docx]

**Figure S6.** Bar graphs of each individual subject’s % change in the following metabolites (subjects ordered by direction and magnitude of response on x axis): A) the arachidonic acid cyclooxygenase metabolite prostaglandin F_2α_ (PGF_2α_); B) the soluble epoxide hydrolase (sEH) eicosapentaenoic acid (EPA, 20:5n3) metabolite 8,9-dihydroxyeicosatrienoic acid (8,9-DiHETE); C) the sEH docosahexaenoic acid (DHA, 22:6n3) metabolite 19,20-dihydroxydocosapentaenoic acid (19,20-DiHDPE); and D) the cytochrome P450 DHA metabolite 10(11)-epoxydocosapentaenoic acid (10(11)-EpDPE).
