# Supplementary material for: Individual Variation in Lipidomic Profiles of Healthy Subjects in Response to Omega-3 Fatty Acids
Source: PLoS One. 2013 Oct 24;8(10):e76575. doi: 10.1371/journal.pone.0076575 (PMC3811983; doi:10.1371/journal.pone.0076575)
Supplement: Table S1 — Dietary record summaries pre vs. post intervention averaged across 5 of the 12 subjects (mean and SD), assessed by paired t-test. (DOCX) [file pone.0076575.s007.docx]

**Table S1.** Dietary record summaries pre vs. post intervention averaged across 5 of the 12 subjects (mean and SD), assessed by paired t-test.

|  | **Pre** | | **Post** | |  |
| --- | --- | --- | --- | --- | --- |
|  | **Mean** | **SD** | **Mean** | **SD** | **p-value** |
| Calories | 2029.40 | 420.17 | 2110.80 | 618.89 | 0.23 |
| Carbohydrates (g) | 288.60 | 83.92 | 256.40 | 73.87 | 0.10 |
| Fat (g) | 71.80 | 15.69 | 74.00 | 17.46 | 0.42 |
| Protein (g) | 79.00 | 26.22 | 82.20 | 28.17 | 0.42 |
| % Carbs | 56 | 9 | 49 | 8 | 0.10 |
| % Fat | 32 | 7 | 32 | 5 | 0.48 |
| % Protein | 16 | 5 | 16 | 6 | 0.45 |
| Fiber (g) | 23.60 | 6.91 | 29.60 | 19.65 | 0.20 |
| Sugars (g) | 129.80 | 70.30 | 104.80 | 36.99 | 0.18 |
| Ca (mg) | 1151.20 | 323.90 | 1073.00 | 321.30 | 0.35 |
| Iron (mg) | 26.00 | 21.41 | 19.60 | 18.12 | 0.27 |
| Vitamin C (mg) | 138.70 | 146.43 | 182.68 | 165.12 | 0.27 |
| Folate (mcg) | 331.80 | 53.84 | 374.40 | 94.71 | 0.16 |
| Choline (mg) | 173.40 | 75.28 | 174.80 | 80.35 | 0.49 |
| Vitamin A (IU) | 6041.00 | 4520.17 | 13911.40 | 14909.64 | 0.09 |
| Vitamin E as α-Tocopherol (mg) | 4.98 | 3.23 | 6.57 | 5.50 | 0.20 |
| Vitamin D (IU) | 254.80 | 161.39 | 183.00 | 112.91 | 0.14 |
| SFA (g) | 28.40 | 9.81 | 26.40 | 8.96 | 0.15 |
| MUFA (g) | 19.40 | 5.55 | 16.78 | 9.41 | 0.25 |
| PUFA (g) | 10.40 | 6.80 | 11.90 | 7.20 | 0.24 |
| 18:2n6 (g) | 3.65 | 2.27 | 0.90 | 1.56 | 0.03 |
| 18:3n3 (g) | 0.29 | 0.30 | 0.19 | 0.25 | 0.32 |
| 20:4n6 (g) | 0.05 | 0.04 | 0.02 | 0.03 | 0.11 |
| 20:5n3 (g) | 0.00 | 0.00 | 0.00 | 0.00 | 0.19 |
| 22:6n3 (g) | 0.00 | 0.01 | 0.00 | 0.00 | 0.35 |
| Alcohol (g) | 4.60 | 10.29 | 22.20 | 32.56 | 0.09 |
| Caffeine (mg) | 63.00 | 37.95 | 76.40 | 79.01 | 0.33 |
