# Supplementary material for: Individual Variation in Lipidomic Profiles of Healthy Subjects in Response to Omega-3 Fatty Acids
Source: PLoS One. 2013 Oct 24;8(10):e76575. doi: 10.1371/journal.pone.0076575 (PMC3811983; doi:10.1371/journal.pone.0076575)
Supplement: Table S2 — Variables that were excluded from analysis because they either had >33% missing variables or were not detected (i.e. were below the limit of quantitation). (DOCX) [file pone.0076575.s008.docx]

**Table S2.** Variables that were excluded from analysis because they either had >33% missing variables or were not detected (i.e. were below the limit of quantitation).

| 5(6)-EpETrE | 11,12-,15-TriHETrE | DG18:4n3 | LY18:4n3 | TGdm18:1n9 |
| --- | --- | --- | --- | --- |
| 8(9)-EpETrE | THF diol | DG20:3n3 | LY20:3n3 | TGt16:1n7 |
| 14(15)-EpETrE | 5,15-DiHETE | DG20:4n3 | LY20:4n3 | TGt18:1n9 |
| 8,9-DiHETrE | 8,15-DiHETE | DG24:6n3 | LY24:6n3 | TGt18:2n6 |
| 8(9)-EpETE | 20-HETE | DGdm16:0 | LYdm18:0 | TGdm |
| 11(12)-EpETE | 8-HEPE | DGdm18:0 | LYdm18:1n7 |  |
| 14(15)-EpETE | CE15:0 | DGdm18:1n7 | LYdm18:1n9 |  |
| 17(18)-EpETE | CE20:1n9 | DGdm18:1n9 | LYt16:1n7 |  |
| 7(8)-EpDPE | CE24:1n9 | DGt16:1n7 | LYt18:1n9 |  |
| 13(14)-EpDPE | CE22:4n6 | DGt18:1n9 | LYt18:2n6 |  |
| 19(20)-EpDPE | CE18:4n3 | DGt18:2n6 | PC18:4n3 |  |
| 4,5-DiHDPE | CE24:6n3 | DGdm | PC20:3n3 |  |
| 7,8-DiHDPE | CEdm16:0 | FFA24:0 | PC24:6n3 |  |
| 5,6-DiHETE | CEdm18:0 | FFA22:2n6 | PCt16:1n7 |  |
| PGE1 | CEdm18:1n7 | FFA18:4n3 | PCt18:1n9 |  |
| PGD1 | CEdm18:1n9 | FFA20:3n3 | PCt18:2n6 |  |
| PGE3 | CEt16:1n7 | FFA24:6n3 | PE14:1n5 |  |
| PGD3 | CEt18:1n9 | FFAdm16:0 | PE22:2n6 |  |
| PGB2 | CEt18:2n6 | FFAdm18:0 | PE18:4n3 |  |
| PGJ2 | CEdm | FFAdm18:1n7 | PE20:3n3 |  |
| 15-deoxy-PGJ2 | DG22:0 | FFAdm18:1n9 | PE24:6n3 |  |
| LXA4 | DG20:1n9 | FFAt16:1n7 | PEt16:1n7 |  |
| LTB3 | DG20:3n9 | FFAt18:1n9 | PEt18:1n9 |  |
| LTB4 | DG24:1n9 | FFAt18:2n6 | PEt18:2n6 |  |
| 6-trans-LTB4 | DG18:3n6 | FFAdm | TG20:3n3 |  |
| LTB5 | DG20:3n6 | LY14:1n5 | TG24:6n3 |  |
| 20-OH-LTB4 | DG22:2n6 | LY24:1n9 | TGdm16:0 |  |
| 20-COOH-LTB4 | DG22:4n6 | LY22:2n6 | TGdm18:0 |  |
| Resolvin E1 | DG22:5n6 | LY22:5n6 | TGdm18:1n7 |  |
