# Supplementary material for: Individual Variation in Lipidomic Profiles of Healthy Subjects in Response to Omega-3 Fatty Acids
Source: PLoS One. 2013 Oct 24;8(10):e76575. doi: 10.1371/journal.pone.0076575 (PMC3811983; doi:10.1371/journal.pone.0076575)
Supplement: Table S3 — Model assessment parameters. (DOCX) [file pone.0076575.s009.docx]

**Table S3.** Model assessment parameters.

| **Plot/Model** | **Data set** | **Number of components** | **Total systematic variation among the X-variables captured by the model (R2X[cum])** | **Total systematic variation among the Y-variables captured by the model (R2Y[cum])** | **Predictive ability of the model Q2[cum]** | **p-values calculated by CV-ANOVA** |
| --- | --- | --- | --- | --- | --- | --- |
| Figure 1A/  OPLS-DA | Lipidomic profile | 1 predictive  2 orthogonal  (1+2+0) | 0.393 | 0.959 | 0.787 | 0.00007 |
| Figure 1B/ OPLS-DA | Lipoprotein profile | 1 predictive  2 orthogonal  (1+2+0) | 0.741 | 0.792 | 0.611 | 0.007 |
| Figure 2/ PCA | De novo lipogenesis variables | 3 | 0.837 |  | 0.681 |  |
| Figure 3A and S5/  OPLS-DA | Oxylipin profile | 1 predictive  (1+0+0) | 0.337 | 0.758 | 0.71 | 0.000002 |
| Figure 3B/  O2PLS | X = oxylipin profile  Y = EPA and DHA profile | 1 joint  1 unique to X  1 unique to Y  (1+1+1) | 0.506 | 0.665 | 0.541 | (Table S6) |
| Figure S2/ PCA | Lipidomic profile | 5 | 0.632 |  | 0.126 |  |
| Figure S3/ PCA | Oxylipin profile | 5 | 0.785 |  | 0.447 |  |
| Figure S4/ PCA | Lipoprotein profile | 3 | 0.761 |  | 0.555 |  |
