# Supplementary material for: Individual Variation in Lipidomic Profiles of Healthy Subjects in Response to Omega-3 Fatty Acids
Source: PLoS One. 2013 Oct 24;8(10):e76575. doi: 10.1371/journal.pone.0076575 (PMC3811983; doi:10.1371/journal.pone.0076575)
Supplement: Table S4 — Individual contributions of each variable to the post profile (high loading values) and pre profile (low loading values). (DOCX) [file pone.0076575.s010.docx]

**Table S4.** Individual contributions of each variable to the post profile (high loading values) and pre profile (low loading values).
