# Supplementary material for: Individual Variation in Lipidomic Profiles of Healthy Subjects in Response to Omega-3 Fatty Acids
Source: PLoS One. 2013 Oct 24;8(10):e76575. doi: 10.1371/journal.pone.0076575 (PMC3811983; doi:10.1371/journal.pone.0076575)
Supplement: Table S5 — The 35 most influential variables to the orthogonal component in the OPLS-DA model (Figure 1a) separating subjects 202 and 220 from the rest of the group. (DOCX) [file pone.0076575.s011.docx]

**Table S5.** The 35 most influential variables to the orthogonal component in the OPLS-DA model (Figure 1a) separating subjects 202 and 220 from the rest of the group.
