# Supplementary material for: Individual Variation in Lipidomic Profiles of Healthy Subjects in Response to Omega-3 Fatty Acids
Source: PLoS One. 2013 Oct 24;8(10):e76575. doi: 10.1371/journal.pone.0076575 (PMC3811983; doi:10.1371/journal.pone.0076575)
Supplement: Table S6 — Additional metabolites that increased or decreased post intervention at α ≤ 0.005. (DOCX) [file pone.0076575.s012.docx]

**Table S6.** Additional metabolites that increased or decreased post intervention at α ≤ 0.005.

| **Metabolite** | **Mean Pre** | **SD Pre** | **Mean Post** | **SD Post** | **Paired t-test** |
| --- | --- | --- | --- | --- | --- |
| Metabolites that increased post intervention | | | | | |
| 12-HEPE (nmol/L) | 33.991 | 35.661 | 195.733 | 136.763 | 0.002 |
| PC22:5n3 (nmol/g) | 32.934 | 8.036 | 51.868 | 13.690 | 0.001 |
| CE22:6n3 (nmol/g) | 18.700 | 5.069 | 36.794 | 9.319 | 0.000 |
| FFA22:6n3 (nmol/g) | 3.452 | 1.586 | 6.453 | 1.939 | 0.002 |
| TG22:4n6 (nmol/g) | 3.420 | 1.212 | 5.060 | 1.823 | 0.001 |
| 19,20-DiHDPE (nmol/L) | 2.760 | 1.043 | 5.194 | 1.833 | 0.0003 |
| CE22:5n3 (nmol/g) | 1.237 | 0.296 | 2.086 | 0.752 | 0.002 |
| FFA22:5n3 (nmol/g) | 0.989 | 0.417 | 1.632 | 0.457 | 0.004 |
| 15-HEPE (nmol/L) | 0.421 | 0.246 | 1.272 | 0.774 | 0.003 |
| DG22:6n3 (nmol/g) | 0.361 | 0.210 | 1.387 | 0.764 | 0.003 |
| 5-HEPE (nmol/L) | 0.233 | 0.138 | 0.674 | 0.378 | 0.002 |
| 13,14-DiHDPE (nmol/L) | 0.184 | 0.054 | 0.333 | 0.110 | 0.0003 |
| TG22:2n6 (nmol/g) | 0.165 | 0.085 | 0.499 | 0.253 | 0.004 |
| 10,11-DiHDPE (nmol/L) | 0.136 | 0.053 | 0.372 | 0.171 | 0.001 |
| Metabolites that decreased post intervention | | | | | |
| PE20:4n6 (nmol/g) | 119.64 | 16.68 | 98.09 | 31.80 | 0.005 |
| 9-HODE (nmol/L) | 114.15 | 55.41 | 66.13 | 18.79 | 0.004 |
| Triglyceride (total) (mg/dL) | 78.58 | 24.92 | 59.87 | 26.11 | 0.0005 |
| VLDL & Chylomicron Particles (total) (nmol/L) | 50.48 | 28.97 | 34.24 | 27.24 | 0.005 |
| VLDL & Chylomicron Triglyceride (total) (mg/dL) | 43.00 | 20.46 | 26.80 | 22.51 | 0.002 |
| 9-oxo-ODE (nmol/L) | 25.60 | 10.21 | 16.27 | 5.09 | 0.004 |
| EKODE (nmol/L) | 23.56 | 12.01 | 9.97 | 5.25 | 0.003 |
| CE20:3n6 (nmol/g) | 23.04 | 8.32 | 15.50 | 4.58 | 0.001 |
| PC20:2n6 (nmol/g) | 14.25 | 3.43 | 10.87 | 3.94 | 0.0003 |
| 13-HOTrE (nmol/L) | 13.14 | 6.24 | 7.75 | 3.10 | 0.005 |
| 9-HOTrE (nmol/L) | 7.66 | 4.08 | 3.14 | 1.34 | 0.001 |
| TG18:3n6 (nmol/g) | 7.46 | 3.86 | 5.72 | 2.92 | 0.004 |
| PC20:3n9 (nmol/g) | 4.63 | 3.25 | 2.25 | 1.25 | 0.003 |
