# Supplementary material for: Individual Variation in Lipidomic Profiles of Healthy Subjects in Response to Omega-3 Fatty Acids
Source: PLoS One. 2013 Oct 24;8(10):e76575. doi: 10.1371/journal.pone.0076575 (PMC3811983; doi:10.1371/journal.pone.0076575)
Supplement: Table S7 — Lipid-specific p-values in the O2PLS model correlating joint variation between oxylipins and EPA and DHA. (DOCX) [file pone.0076575.s013.docx]

**Table S7**. Lipid-specific p-values in the O2PLS model correlating joint variation between oxylipins and EPA and DHA.

| **Lipid** | **p-value** |
| --- | --- |
| LY20:5n3 | 1.67E-06 |
| PC20:5n3 | 4.39E-06 |
| FFA22:6n3 | 2.35E-05 |
| CE20:5n3 | 6.73E-05 |
| TG20:5n3 | 0.000128 |
| PE20:5n3 | 0.000363 |
| PC22:6n3 | 0.001843 |
| CE22:6n3 | 0.00333 |
| FFA20:5n3 | 0.003691 |
| TG22:6n3 | 0.005754 |
| LY22:6n3 | 0.062918 |
| DG22:6n3 | 0.113673 |
| PE22:6n3 | 0.751191 |
| DG20:5n3 | 0.812763 |
