# Supplementary material for: Individual Variation in Lipidomic Profiles of Healthy Subjects in Response to Omega-3 Fatty Acids
Source: PLoS One. 2013 Oct 24;8(10):e76575. doi: 10.1371/journal.pone.0076575 (PMC3811983; doi:10.1371/journal.pone.0076575)
Supplement: Table S8 — Variables with joint variation according to O2PLS-DA modeling (the higher loading value, the more influential the variable). (DOCX) [file pone.0076575.s014.docx]

**Table S8.** Variables with joint variation according to O2PLS-DA modeling (the higher loading value, the more influential the variable).
