# Supplementary material for: Individual Variation in Lipidomic Profiles of Healthy Subjects in Response to Omega-3 Fatty Acids
Source: PLoS One. 2013 Oct 24;8(10):e76575. doi: 10.1371/journal.pone.0076575 (PMC3811983; doi:10.1371/journal.pone.0076575)
Supplement: Table S9 — Variables among the oxylipins with unique variation (and little or no correlation with EPA and DHA) according to O2PLS-DA modeling (the lower the value, the more influential). (DOCX) [file pone.0076575.s015.docx]

**Table S9.** Variables among the oxylipins with unique variation (and little or no correlation with EPA and DHA) according to O2PLS-DA modeling (the lower the value, the more influential).
