# Supplementary material for: Individual Variation in Lipidomic Profiles of Healthy Subjects in Response to Omega-3 Fatty Acids
Source: PLoS One. 2013 Oct 24;8(10):e76575. doi: 10.1371/journal.pone.0076575 (PMC3811983; doi:10.1371/journal.pone.0076575)
Supplement: Table S10 — Results from standard lease squares linear regressions for each variable adjusted for age, gender, body weight, and BMI, with p-values ≤ 0.05. (DOCX) [file pone.0076575.s016.docx]

**Table S10.** Results from standard lease squares linear regressions for each variable adjusted for age, gender, body weight, and BMI, with p-values ≤ 0.05.

| **Variable** | **Term** | **Estimate** | **Std Error** | **t Ratio** | **Prob>\|t\|** | **Prob > F** | **Rsquare** |
| --- | --- | --- | --- | --- | --- | --- | --- |
| % Change CE18:1n9 | Intercept | 101.9469406 | 46.84410831 | 2.176302299 | 0.07 | 0.03 | 0.786 |
|  | Age | -1.666922292 | 0.452218824 | -3.686096649 | 0.01 |  |  |
|  | Gender[Female] | -21.98641963 | 6.166521934 | -3.565449027 | 0.01 |  |  |
|  | BMI | 3.035512549 | 2.642655468 | 1.14865997 | 0.29 |  |  |
|  | Body Weight (kg) | -1.731317487 | 0.536395537 | -3.227688094 | 0.02 |  |  |
| % Change Large HDL Particles | Intercept | 21.00609115 | 76.66358596 | 0.274003504 | 0.79 | 0.01 | 0.796 |
|  | Age | 2.116718022 | 0.811086788 | 2.609730615 | 0.03 |  |  |
|  | Gender[Female] | -20.58397849 | 11.15289548 | -1.845617447 | 0.11 |  |  |
|  | BMI | 3.528346474 | 4.469022225 | 0.789511955 | 0.46 |  |  |
|  | Body Weight (kg) | -2.279397669 | 0.963656938 | -2.365362173 | 0.05 |  |  |
| % Change PC18:0 | Intercept | 36.95354092 | 23.41463605 | 1.578224015 | 0.16 | 0.002 | 0.897 |
|  | Age | -1.186182837 | 0.247722588 | -4.78835154 | 0.002 |  |  |
|  | Gender[Female] | -20.26496507 | 3.406323684 | -5.949218852 | 0.001 |  |  |
|  | BMI | 6.180668863 | 1.364931311 | 4.528190404 | 0.003 |  |  |
|  | Body Weight (kg) | -2.061326793 | 0.29432065 | -7.00367708 | 0.0002 |  |  |
| % Change PC Total FA | Intercept | 47.11789576 | 22.69417603 | 2.076210905 | 0.08 | 0.01 | 0.813 |
|  | Age | -0.857991242 | 0.240100252 | -3.573470803 | 0.01 |  |  |
|  | Gender[Female] | -16.29927151 | 3.301512317 | -4.936910707 | 0.002 |  |  |
|  | BMI | 2.901205617 | 1.32293286 | 2.193010472 | 0.06 |  |  |
|  | Body Weight (kg) | -1.280008749 | 0.285264509 | -4.487094294 | 0.003 |  |  |
| % Change PEdm18:1n7 | Intercept | 253.9315287 | 108.2878532 | 2.344967799 | 0.05 | 0.01 | 0.805 |
|  | Age | 1.784685373 | 1.14566578 | 1.557771389 | 0.16 |  |  |
|  | Gender[Female] | -42.58166437 | 15.75354313 | -2.702989671 | 0.03 |  |  |
|  | BMI | -12.28341094 | 6.312525255 | -1.945879096 | 0.09 |  |  |
|  | Body Weight (kg) | -0.18236706 | 1.361172188 | -0.133977951 | 0.90 |  |  |
| % Change TG22:0 | Intercept | -181.2638604 | 113.05623 | -1.603307136 | 0.17 | 0.01 | 0.893 |
|  | Age | -0.335840158 | 1.2379815 | -0.271280433 | 0.80 |  |  |
|  | Gender[Female] | 34.38823637 | 18.46894696 | 1.861948948 | 0.12 |  |  |
|  | BMI | 19.64536152 | 7.504266274 | 2.617892383 | 0.05 |  |  |
|  | Body Weight (kg) | -3.881250723 | 1.703746308 | -2.278068457 | 0.07 |  |  |
