# Supplementary material for: Individual Variation in Lipidomic Profiles of Healthy Subjects in Response to Omega-3 Fatty Acids
Source: PLoS One. 2013 Oct 24;8(10):e76575. doi: 10.1371/journal.pone.0076575 (PMC3811983; doi:10.1371/journal.pone.0076575)
Supplement: Table S11 — Gender differences in response to treatment. Mean and Standard Error (SE) for females vs. males compared by t-test. (DOCX) [file pone.0076575.s017.docx]

**Table S11.** Gender differences in response to treatment. Mean and Standard Error (SE) for females vs. males compared by t-test.

|  | **Females** | | **Males** | |  |
| --- | --- | --- | --- | --- | --- |
| **Metabolite** | **Mean** | **SE** | **Mean** | **SE** | **P-value** |
| % change 11(12)-EpETrE | 39.6 | 26.3 | -19.9 | 8.43 | 0.03 |
| % change 11,12-DiHETrE | -9.84 | 6.48 | -36.1 | 5.15 | 0.005 |
| % change 14,15-DiHETrE | -10.3 | 7.17 | -31.1 | 4.26 | 0.02 |
| % change 8,9-DiHETE | 818 | 128 | 296 | 93.8 | 0.004 |
| % change 11,12-DiHETE | 1595 | 573 | 187 | 25.8 | 0.02 |
| % change 14,15-DiHETE | 987 | 204 | 296 | 68.4 | 0.006 |
| % change 17,18-DiHETE | 701 | 195 | 238 | 51.6 | 0.03 |
| % change 10,11-DiHDPE | 297 | 74.2 | 96.2 | 67.8 | 0.04 |
| % change 13,14-DiHDPE | 110 | 25.2 | 42.9 | 16.0 | 0.02 |
| % change 16,17-DiHDPE | 137 | 29.2 | 52.9 | 14.3 | 0.01 |
| % change 19,20-DiHDPE | 120 | 25.5 | 49.5 | 14.0 | 0.02 |
| % change 15-HEPE | 494 | 177 | 110 | 67.0 | 0.04 |
| Weight-Adjusted EPA Dose | 26.5 | 1.39 | 18.1 | 1.72 | 0.003 |
| Weight-Adjusted DHA Dose | 33.7 | 1.77 | 23.0 | 2.19 | 0.003 |
| % change Large VLDL & Chylomicrons Particles | -71.0 | 8.07 | -89.4 | 5.15 | 0.04 |
| % change Medium VLDL Particles | -42.4 | 25.1 | 33.7 | 22.4 | 0.03 |
| % change Medium Small LDL Particles | 155 | 64.9 | 5.4 | 12.5 | 0.03 |
| % change VLDL Size (nm) | 0.63 | 6.22 | -15.2 | 3.46 | 0.03 |
| % change DG20:5n3 | 1739 | 668 | 219 | 46.0 | 0.04 |
| % change DG22:6n3 | 681 | 193 | 96.5 | 65.4 | 0.01 |
| % change FFA18:0 | -4.84 | 11.6 | -29.1 | 6.56 | 0.05 |
| % change FFA22:0 | 122 | 75.1 | -31.3 | 19.9 | 0.04 |
| % change FFA18:3n6 | 35.4 | 38.6 | -55.1 | 10.9 | 0.03 |
| % change FFA20:4n6 | 11.3 | 15.5 | -28.9 | 9.45 | 0.03 |
| % change FFA20:5n3 | 720 | 200 | 119 | 106 | 0.01 |
| % change PE22:0 | -1.24 | 15.5 | 100 | 29.7 | 0.02 |
| % change PE22:5n6 | -58.9 | 5.87 | -43.3 | 2.31 | 0.02 |
| % change TG22:0 | 50.6 | 14.6 | -48.4 | 11.5 | 0.0004 |
| % change TG20:1n9 | 59.4 | 21.6 | -13.3 | 6.80 | 0.006 |
| % change TG22:1n9 | 34.1 | 18.6 | -18.9 | 17.8 | 0.04 |
| % change TG22:4n6 | 73.3 | 20.8 | 24.6 | 16.1 | 0.05 |
| Baseline PC ARA% | 9.21 | 0.82 | 11.1 | 0.48 | 0.04 |
